# Systematic review of the epidemiological evidence of associations between quantified occupational exposure to respirable crystalline silica and the risk of silicosis and lung cancer

**DOI:** 10.3389/fpubh.2025.1554006

**Published:** 2025-02-28

**Authors:** Kenneth A. Mundt, William J. Thompson, Gaurav Dhawan, Harvey Checkoway, Paolo Boffetta

**Affiliations:** ^1^Epidemiology, University of Massachusetts, Amherst, MA, United States; ^2^Independent Consultant, Hume, MO, United States; ^3^Sri Guru Ram Das (SGRD), University of Health Sciences, Amritsar, India; ^4^Independent Consultant, Hartford, CT, United States; ^5^Division of Climate and Environmental Health, Herbert Wertheim School of Public Health, University of California, San Diego, San Diego, CA, United States; ^6^Department of Medical and Surgical Sciences, University of Bologna, Bologna, Italy; ^7^Stony Brook Cancer Center, Stony Brook University, Stony Brook, NY, United States; ^8^Department of Family, Population and Preventive Medicine, Renaissance School of Medicine, Stony Brook University, Stony Brook, NY, United States

**Keywords:** crystalline silica, silicosis, lung cancer, systematic review, exposure-response, epidemiology

## Abstract

**Introduction:**

Occupational exposure to respirable crystalline silica (RCS) has been associated with both silicosis and lung cancer, but no systematic review (SR) specifically focused on exposure-response relationships has been published for these diseases.

**Methods:**

We conducted this SR in compliance with Preferred Reporting Items for Systematic reviews and Meta-Analyses (PRISMA) guidelines. PubMed searches, supplemented with Web of Science and Google Scholar searches, identified 1,007 potentially relevant articles. After applying selection criteria and removing duplicates, 65 publications were reviewed and evaluated, 20 of which presented at least semi-quantitative exposure-response results for lung cancer (*n* = 12) and/or silicosis (*n* = 10).

**Results:**

Cumulative RCS exposure was most commonly reported. Increasing silicosis risk with increasing cumulative RCS exposure was reported in all studies, with exposure thresholds indicated, but at different cumulative exposures. For most studies defining silicosis as International Labor Organization (ILO) score ≥ 1/0, substantially increased risks were clear at or above 1 mg-/m^3^-yr. For lung cancer, exposure-response estimates were mixed with 4 studies reporting no statistically significantly increased relative risk of lung cancer at any cumulative RCS exposure. Three studies reported statistically significant increased risks but only for high cumulative RCS exposures. Residual confounding by smoking was not explicitly discussed in most studies. One case–control study presented an exposure-response analysis for silica and lung cancer limited to never-smokers with substantial silica exposure; risk was increased only among those in the highest RCS exposure category. Studies with more detailed smoking information generally reported risks close to background levels except at the highest cumulative RCS exposure categories.

**Conclusion:**

Silicosis risk clearly and consistently was increased above cumulative exposure thresholds of roughly 1 mg/m^3^-years across most studies. However, for lung cancer, results were heterogeneous with potential residual confounding by smoking complicating interpretation. Results suggest that lung cancer risk may not be increased at cumulative RCS exposures below the reported exposure thresholds for silicosis risk.

## 1 Introduction

Crystalline silica occurs naturally in rock, stone, soils, and sands. It is a critical component of infrastructure, construction and building products. Occupational activities including cutting, drilling, chipping, crushing, sanding, or grinding materials containing substantial proportions of quartz, or crystalline silica, can release respirable crystalline silica (RCS) particles, especially absent proper controls, although very low concentrations also can be detected in ambient air (1). Respirable crystalline silica (RCS) particles are defined as those less than 4 μm in aerodynamic diameter. Inhalation of sufficient quantities of RCS increases the risk of several respiratory conditions and diseases including silicosis and probably lung cancers (1).

Silicosis is a pulmonary disease characterized by inflammation and subsequent development of silicotic nodules (i.e., fibrosis) in the lungs, with clinical subtypes including acute, accelerated, and chronic - simple or nodular, and chronic—complicated (2). However, epidemiological studies rarely identify subtypes, and traditionally classified silicosis according to the 12-point scale (0/0, 0/1, 1/0... to 3/3+) developed by the International Labor Organization (ILO) for classifying silicosis based on the reading (by at least two independent trained and blinded radiologists) of chest radiographs for epidemiological research purposes (3, 4). Historically, silicosis was most often defined as ILO scores of 1/1 or higher; however, in some countries and especially more recently, ILO scores of 1/0 and higher are considered positive indicators of silicosis (5, 6).

Lung cancers arise from malignant cells in the lungs with uncontrolled growth. Although there are several types of lung cancers, they often are categorized into two major groups, small cell lung cancers (SCLC) and non-small cell lung cancers (NSCLC), the latter being the most common form and including adenocarcinoma, squamous cell carcinoma, large-cell undifferentiated carcinoma, adenosquamous carcinoma, and sarcomatoid carcinoma (7, 8). Tobacco smoking is the most prevalent preventable cause of lung cancer of all common types (9).

IARC (International Agency for Research on Cancer) (10) classified RCS as a Group 1 human carcinogen, concluding that they found “sufficient evidence in humans for the carcinogenicity of inhaled crystalline silica in the form of quartz or cristobalite from occupational sources” but also clarified: “ln making the overall evaluation, the Working Group noted that carcinogenicity in humans was not detected in all industrial circumstances studied. Carcinogenicity may be dependent on inherent characteristics of the crystalline silica or on external factors affecting its biological activity or distribution of its polymorphs” (10). Increased risk of lung cancer has been reported in both animal and epidemiological studies, indicating that lung cancer risk likely is increased at exposure levels that also cause silicosis. It has been hypothesized that acute inflammation caused by high concentrations of RCS induces an immunosuppressive microenvironment that induces fibrosis and promotes tumor growth (2, 11).

Numerous epidemiological investigations have demonstrated clear relationships between heavy historical occupational RCS exposure and increased risk of silicosis—and in some studies lung cancers—often among workers historically employed in mining, quarrying, manufacturing of building materials, some construction trades, production of pottery and porcelain wares [e.g., (12–16)]. However, the RCS exposure scenarios epidemiologically associated with increased risks of either of these serious pulmonary diseases generally are seen with exposure intensities and cumulative exposures that are orders of magnitude higher than those resulting from ambient respirable silica exposure concentrations, even over a lifetime.

Furthermore, while many studies have reported results suggesting exposure thresholds for risk of silicosis and lung cancer, efforts to quantify exposure thresholds have not been straightforward or consistent, and there are few examples from the published literature where study objectives included or exclusively addressed defining the exposure threshold for silicosis [e.g., (17–19)]. Some studies assume linear no-threshold exposure-response relationships [e.g., (12, 20)]. Despite uncertainties surrounding the RCS exposure levels associated with increased risk of silicosis or lung cancers, policymakers, risk assessors, experts in litigation—among others—have relied on select reported study results suggesting that exposure to low concentrations of RCS increases the risk of nonmalignant respiratory diseases including silicosis, and lung cancers. This highlights the importance of and need for an objective and transparent systematic review of scientific epidemiological literature that plainly sets forth the best epidemiological evidence and objective interpretations on which to base conclusions and policy decisions regarding risks associated with ambient to low non-occupational RCS exposures. Identifying the levels at which risks of pulmonary diseases such as silicosis and lung cancers increase can inform policy and support effective preventive actions.

Therefore, the primary aim of this systematic review (SR) was to elucidate under what exposure settings, and more specifically at what quantified levels of RCS exposure risk of silicosis and lung cancer is observed to be measurably increased. This is addressed by applying systematic SR principles and methods to evaluate the epidemiological literature quantitatively (or at least semi-quantitatively) exploring exposure-response relationships between RCS exposure and the risk of silicosis and lung cancer, respectively, including identifying and quantifying potential exposure thresholds for such risks.

## 2 Methods

This SR employed a hybrid framework that incorporates elements proposed and advocated by the National Toxicology Program (NTP) Office of Health Assessment and Translation (OHAT), the Integrated Risk Information System (IRIS), the Toxic Substances Control Act (TSCA) and the Office of Pollution Prevention and Toxics (OPPTS), respectively, that also aligned with guidance from the US National Academies of Sciences, Engineering, and Medicine (NASEM). This approach previously has been described and applied in other settings [e.g., (21, 22)].

Protocol development and review reporting were guided by the Preferred Reporting Items for Systematic Reviews and Meta-Analyses (PRISMA) checklist (23). The SR protocol was registered with PROSPERO (registration identification CRD 42023468033 in October 2023).

### 2.1 Study search methods

Searches of the peer-reviewed published medical and health literature were conducted primarily using PubMed and supplemented by searches of Web of Science and Google Scholar. To identify studies that quantitatively evaluated the associations between respirable silica and risk of silicosis or lung cancer in occupational settings—or exceptionally, in the general population—the following specific search terms were applied: ((silica*[Title]) AND (lung[Title] OR silicosis[Title] OR mortality[Title] OR morbidity[Title] OR disease*[Title]) AND (exposure[Title/Abstract] OR risk[Title/Abstract]) AND (worker*[Title/Abstract] OR occupation*[Title/Abstract] OR mine*[Title/Abstract] OR cohort[Title/Abstract])). Two reviewers (WT, GD) independently evaluated titles and abstracts for general relevance and adherence to Population, Exposure, Comparator and Outcome (PECO) criteria (24). Any disagreements were discussed and if necessary, adjudicated by a third independent reviewer (KM).

### 2.2 Screening, results abstraction and evaluation

**Figure 1:**
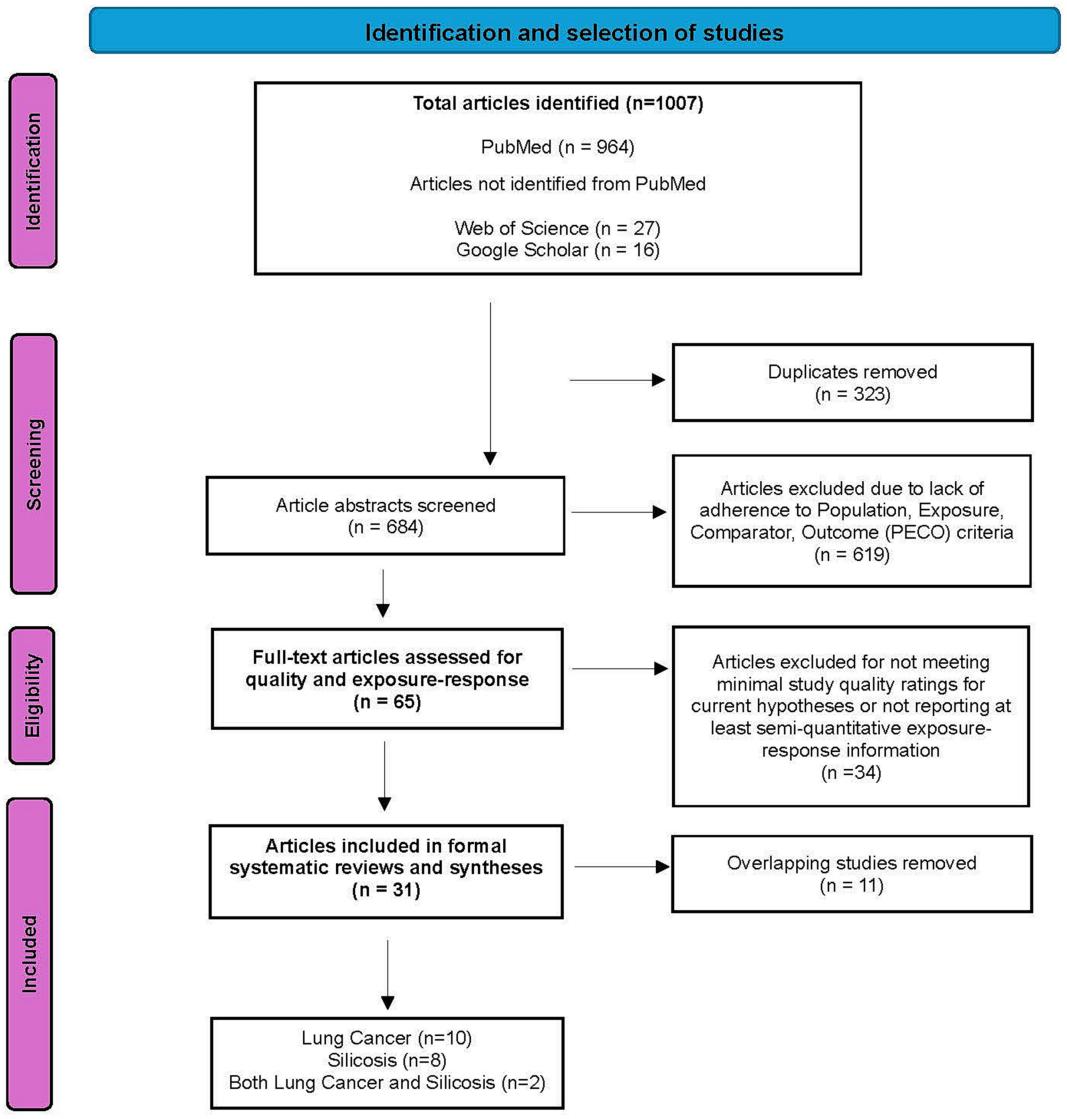
Flow diagram of the study selection process for the systematic review of silica exposure and risk of lung cancer and/or silicosis [Adapted from: Page et al. (23)].

A summary of the study selection process is presented in Figure 1, a PRISMA flow diagram. We identified 1,007 articles in total, of which 964 were from PubMed searches. An additional 27 and 16 articles that had not been identified from the PubMed searches were identified from searches of Web of Science and Google Scholar, respectively. Upon initial screening of the 1,007 references, we identified and removed 323 duplicate records, and then evaluated the remaining 684 articles as follows. Screening of the abstracts of these identified 619 articles that did not adhere to the PECO criteria and were excluded (list of excluded papers available upon request). For each of the 65 articles meeting the inclusion criteria, full texts were obtained, and data were extracted into an Excel table. Results from each selected and verified eligible epidemiological study were reviewed and evaluated to provide a basis for rating study quality for evidence synthesis and weighting, including study participation, exposure characterization, outcome assessment, potential confounding/variability control, and analysis, according to the SR objectives and hypotheses. Key studies were tabulated by one reviewer and verified/quality-controlled by a second.

Based on the extracted data, studies were further evaluated for quality applying a four-tiered approach where those of lowest quality for evaluating our hypotheses were eliminated at Tier 1 (see Supplementary Figure S1). Studies that were excluded at Tier 1, regardless of the quality ratings for other study aspects, typically did not report quantitative exposure metrics, as our key systematic review hypotheses hinged on quantified exposure and exposure-response assessments. Those that minimally (i.e., at least semi-quantitatively) quantified exposure or exposure categories were further evaluated at Tier 2 based on whether adequate control of confounders (e.g., age and smoking) was documented. This was especially important for studies reporting relationships between quantified silica exposure and risk of lung cancer. However, we did not exclude studies that did not consider other occupational exposures as potential confounders but noted where results controlled for any of these. In total (i.e., across Tiers 1 and 2), 34 articles failed to meet minimal quality expectations for control of confounding (especially tobacco smoking for lung cancer) or did not report exposure-response results or provide data allowing *post hoc* assessment. Studies of sufficient quality entering Tier 3 were then evaluated based on the quality of participant selection, outcome measurements, additional necessary statistical adjustments, as well as the overall study design and methodology employed. Studies were classified as “high quality” if most of these criteria adequately or fully were met. Studies that otherwise were of good quality but were considered to have important limitations were classified as “medium quality.” Finally, for Tier 4, all 31 “medium quality” and “high quality” studies were assessed and summarized according to the exposure-response information available.

### 2.3 Systematic review and synthesis

The 31 articles presenting at least semi-quantitative exposure-response results for at least one of these two outcomes or reporting necessary information that allowed deriving exposure-responses with them, were systematically reviewed. Substantial overlap was identified in a subset of the articles resulting in the exclusion of 11 studies (13, 14, 25–32, 33). After removing the overlapping studies, relevant findings were available for the following subsets of articles: lung cancer only (*n* = 10), silicosis only (*n* = 8), or both (*n* = 2). Most studies were rated medium quality based on the domains of participant selection, outcome assessment, evaluation of potential confounding and analysis (see heat maps for quality ratings by domain in Supplementary Figures S2, S3). Exposure-response results from these studies addressing lung cancer (*n* = 12) and silicosis (*n* = 10) are presented in separate tables, allowing comparisons of each study based on study design features and quality assessment with all others for each health outcome, and quality-weighted synthesis of the body of evidence. Specific strengths and limitations of the body of systematically reviewed studies as well as critical evidentiary gaps were identified and discussed.

## 3 Results

### 3.1 Silicosis studies

Table 1 summarizes the 10 studies included for systematic review, seven of which were cohort studies, two were nested case–control studies, and one was a cross-sectional study. Four studies were conducted in the United States (US), two in China, and one each in Germany, Spain, Sweden and the United Kingdom (UK). Six studies addressed RCS exposures in mining and quarrying and five in other settings (one study included both miners and non-miners). The number of silicosis cases in these studies ranged from as few as six (34) to as many as 9,377 (15); however, no individual study conducted on workers outside of China reported more than 81 silicosis cases. Note that Wang et al. (15) is the most recent update on silicosis of the combined cohorts of Chinese workers in multiple industries but has substantial overlap with three others (13, 26, 27), which were excluded.

**Table 1:** Description of studies with exposure-response for silica and risk of silicosis.

Study (First author year)	Study design	Industry	Country	Earliest employment or year studied	Adjustment variables	Overall # of cases	Exposure Assessment Method	Outcome assessment method
Cherry 1998	Cohort	Pottery, refractory, and sandstone industries	UK	1929	Smoking (ever/never)	64	JEM based on personal and area sampling	Chest radiograph based on ILO rating ≥ 1/0
Hughes 1998 (overlaps with Park 2002)	Cohort	Diatomaceous earth mining and processing	US	1942	Age	81	Job category estimates based on air monitoring and relative rankings	Chest radiograph based on ILO rating ≥ 1/0
Steenland 2001	Cohort	Sand Industry	US	1946	Smoking (limited data available)	14	JEM based on personal and area sampling	Death certificates, silicosis and unspecified pneumoconiosis (ICD-9 code 502 and 505)
Rego 2008	Cross-sectional	Granite Industry	Spain	2004	Smoking, Age	77	Job categories based on area sampling	Chest radiograph based on ILO rating ≥ 1/1 and round opacities
Zhang 2010	Cohort	Automobile foundry	China	1980	Employment length (yrs), smoking, age, gender, alcohol, pulmonary tuberculosis	48	Employment history and air monitoring samples	Diagnosis using China’s National Diagnostic Criteria for Silicosis
Vacek 2011	Nested Case–Control	Granite Industry	US	1947	Smoking (limited information available)	55	JEM based on area sampling	Death certificates, silicosis (ICD-9 code 502)
Vacek 2019 (overlaps with Hughes 2001; McDonald 2005)	Nested Case–Control	Sand Industry	US	1938	Respirator use; age	67	JEM based on personal sampling	Chest radiograph based on ILO rating ≥ 1/0
Wang 2020a (overlaps with Lai 2018; Chen 2012; Chen 2001; Chen 1992)	Cohort	Metal mines and pottery factories	China	1960	Smoking, sex, year at hire, age at hire, type of facility (tungsten mine, iron and/or copper mine, tin mine, and pottery factory)	9,377	JEM based on area sampling	Chest radiograph based on China Stage I (similar to ILO rating ≥ 1/0)
Lenander-Ramirez 2022	Cohort	Iron Foundries	Sweden	1913	None reported	6	Job categories based on area and personal sampling	Medical record system (ICD-10 code J62)
Birk 2025 (overlaps with Mundt 2011)	Cohort	Porcelain manufacturing	Germany	1938	Smoking, age, sex, duration of employment	156	JEM based on personal and area sampling	Chest radiograph based on ILO rating ≥ 1/0

Four studies classified radiographic evidence for silicosis using the ILO score ≥ 1/0 (38, 40, 43, 44) and one used an ILO score ≥ 1/1 (35). Three studies only referred to death certificates or medical records for the diagnosis of silicosis (34, 37, 39). The two studies from China (15, 36) used China’s National Diagnostic Criteria for Silicosis and only one (15) described the specific staging as being similar to ILO score ≥ 1/0.

Studies meeting at least minimal quality assessment standards are summarized below, presented by industrial setting.

#### 3.1.1 Mining and quarrying

Spanish granite workers. Rego et al. (35) conducted a cross-sectional study over 2004–2005 of 440 workers employed for a year or more in the granite industry in Pontevedra, Spain. Silicosis diagnosis was based on two independent readings of chest radiographs demonstrating round opacities and using an ILO score > 1/1, and risks were evaluated relative to cumulative silica exposure and employment duration (35). Quantitative silica exposure was estimated using area sampling based on job categories. Logistic regression evaluated silicosis risk by quintiles of cumulative silica exposure adjusting for age; however, the first two quintiles were combined as the reference group, as the first exposure quintile had no silicosis cases. Overall, 77 cases of silicosis were identified. Smoking (pack years) was included in the model with silica exposure as a continuous variable. Notable limitations include the cross-sectional design as well unknown silica exposure prior to 1991 due to lack of regulation and ineffective exposure controls in that industry in Spain (35).

US granite workers. Vacek et al. (37) conducted a case–control study nested in a cohort of 7,052 men employed in the Vermont (US) granite industry between 1947 and 1998. The 55 silicosis deaths identified through 2004 primarily were based on the US National Death Index (NDI), and 162 controls randomly density sampled based on birth year and known to alive at the time of each silicosis death. Quantitative silica exposure assessment was based on a job exposure matrix linked to job histories (22 categories) and 5,204 exposure measurements taken between 1924 and 2004. Quintiles of cumulative silica exposure (in mg/m^3^-yrs) were defined as ≤1.04, 1.05–3.64, 3.65–6.71. 6.72–10.21, and > 10.21, respectively. Individual smoking data were unavailable, although a survey of a subset of the cohort participating in a lung function study reported 50% smoking prevalence compared to the US prevalence of 37% at the time. Conditional logistic regression evaluated the relationships, respectively, between silicosis mortality and net exposure duration, cumulative exposure and average exposure. The number of silicosis deaths was reasonably large, and the quantitative exposure assessment allowed exposure-response patterns; however, only silicosis mortality was assessed.

US diatomaceous earth workers. The mortality and silicosis morbidity of a cohort of 2,342 men employed at a diatomaceous earth plant in California (US) were evaluated and updated in multiple publications, two of which presented detailed exposure-response analyses for silicosis (32, 38). Both are included here as each presented different analyses and information of interest. Chest radiographs were performed since the 1930s as part of a health surveillance program and workers with opacities and ILO score ≥ 1/0 were defined as having silicosis. Although 81 silicosis cases were noted by Hughes et al. (38), Park et al. (32) excluded silicosis cases with onset prior to 1942 or within 1 year of starting employment, resulting in an analysis set of 70 silicosis cases. Quantitative RCS exposure estimates were based on job category air monitoring and relative exposure ranking of jobs. Hughes et al. (38) used Poisson regression to derive relative age-adjusted risk estimates for categories of higher exposure (>1– < 3; >3– < 6; and > 6 mg/m^3^-yrs) compared to the lowest exposure category (< 1 mg/m^3^-yrs). Results were separately reported for all concentrations, concentrations <0.50 mg/m^3^ and > 0.50 mg/m^3^. Park et al. (32) reanalyzed the silicosis data using Poisson regression, and included internal and external adjustments for calendar time, age, smoking, Hispanic ethnicity, and time since first observation while restricting to observations with less than 10 mg/m^3^-yr. They also used various models to determine the best-fitting exposure-response curve. Overall, this cohort provides important information on the exposure-response for silica reported as primarily cristobalite.

US industrial sand workers. Steenland and Sanderson (39) conducted a cohort mortality study of 4,626 industrial sand workers employed at least 1 week at one of 18 plants in 11 US states. The cohort was enumerated in 1987–1988 and followed for mortality through 1996. Although the study focused on lung cancer, mortality due to “silicosis and unspecified pneumoconiosis” also was evaluated and reported. Quantitative silica exposure was estimated using a job exposure matrix based on 4,269 industrial hygiene measurements collected between 1974 and 1995. Quartiles of cumulative respirable silica exposure analyzed were > 0- ≤ 0.10, >0.10–0.51, >0.51–1.28, and > 1.28 mg/m^3^-yrs, respectively. SMRs were calculated using US reference rates and SRRs generated using the lowest quartile as the reference group. Individual information on smoking was unavailable and therefore not used in the silicosis analysis, although smoking was assessed for a cross-sectional sample of 404 men in the cohort and used for indirect comparisons in the lung cancer analyses (see lung cancer section below). Study limitations include the use of death certificates to identify silicosis, of which there were only 14 deaths.

Vacek et al. (40) conducted a case–control study nested in a cohort of 1,902 men employed >10 years between 1938 and 2003 at two US industrial sand companies encompassing 40 different plants in 22 states. Prior publications of this cohort reported increased risk of silicosis by cumulative (OR = 1.43 per 1 mg/m^3^-yrs, 95% CI: 1.23–1.66) and average exposure (OR = 1.30 per = 0.10 mg/m^3^, 95% CI: 1.11–1.51) as well as duration of exposure (OR = 1.10 per year, 95% CI: 1.05–1.16) (30, 31, 41). For silicosis, 67 cases were identified based on the most recent chest radiograph with ILO score > 1/0 based on at least two of the three certified B-reader evaluations. Three controls per case were randomly selected from among men employed at the same plant who were born within 3 years of the case and without an ILO score ≥ 1/0 prior to the date of the case’s earliest radiograph meeting this criterion. The final statistical analysis set had 67 cases and 167 controls. Quantitative silica exposure assessment was derived using a JEM based on work histories and personal exposure monitoring samples. Conditional logistic regression analysis was performed, adjusting for age and respirator use. Univariate and multivariate exposure-response analyses were evaluated by cumulative exposure, average concentration or net duration of exposure. Overall, this study had good ascertainment of silicosis cases and reasonable quantitative exposure estimates based on a JEM.

Chinese metal miners. Wang et al. (15) reported on a cohort of 39,808 workers employed at least 1 year between 1960 and 1974 in any of 20 metal (tungsten, tin, iron/copper) mines or 9 pottery factories in central and southern China and followed through 2003 (1.15 million person-years at risk). Prior publications on this cohort addressing silicosis include Lai et al. (13), Chen et al. (26), Chen et al. (27) and Chen et al. (28). The study used “stage I” or higher silicosis based on the Chinese national diagnostic criteria, reported as having an 89.3% agreement with ILO score 1/0. Nearly a quarter of the cohort (9,377 cases) developed silicosis. Workers with silica exposure had a chest radiograph every 1 or 2 years while working and once every 2 to 4 years after exposure ceased. Quantitative assessment of silica exposure was obtained using a JEM linked to job histories from employment files and an area industrial hygiene sampling scheme. The mean respirable silica dust concentration for each worker was obtained by dividing cumulative respirable silica dust by the net years in a dusty job. Cox proportional hazard analysis assessed quantitative exposure-response adjusting for sex, year of hire, and type of facility. Cumulative dust exposure was categorized into quartiles based on exposure distribution percentiles as follows: 0–0.87; 0.88–2.24; 2.25–5.50 and > 5.50 mg/m^3^-yr, respectively. Silicosis relative risks were consistently higher among smokers than among never-smokers. The study benefits from a large cohort with a high occurrence of silicosis and detailed smoking information but is limited by the exclusion of over 8,000 workers (approximately 25% of the cohort) with missing work histories and missing smoking information.

#### 3.1.2 Ceramics and porcelain industries

Chinese pottery workers. Wang et al. (15) as noted above included 8,883 workers in nine pottery factories. The 1,215 silicosis cases were evaluated according to the same cumulative silica exposure categories used for the miners. Results for pottery workers were compared with those of metal miners and risks were reported to be lower among pottery workers for comparable cumulative exposure levels.

British pottery workers. The mortality of a cohort of pottery workers in Stoke-on-Trent in England was analyzed and reported in two publications (42, 43). Cherry et al. (42) did not report exposure-response results for silicosis. Cherry et al. (43) analyzed a sub-cohort of 1,066 workers with at least 10 years employment in the potteries before 1960 “to test whether exposure estimates were related to radiographic changes in a reasonable way.” Unconditional logistic regression, with adjustment for ever/never smoking, was used to separately evaluate cumulative exposure, duration and average RCS concentration for the 64 cases with ILO chest radiographs scores ≥1/0. Quantitative assessment of silica exposure was conducted by using a job exposure matrix constructed from both personal and area sampling and linked to job histories. This study had adequate exposure information and silicosis diagnosis based on periodic chest radiograph readings but is limited for elucidating the full spectrum of exposure-response relationships due to the study limiting exposure estimates to time periods when exposure was believed to be highest.

German porcelain workers. Mundt et al. (14) described the initial results of the German Porcelain Workers Cohort Study, a cohort of 17,644 employees of 6 months or longer at any of more than 100 porcelain-manufacturing plants in Germany enrolling in a medical screening program during January 1, 1985, through December 31, 1987. Birk et al., (44) updated this cohort through 2020, adding 15 more years of follow-up and generating a total of 537,129 person-years (a 59% increase). Silicosis cases (*n* = 40) in the original study were defined based on a consensus B-reading of chest radiographs with evidence of small, rounded opacities and an ILO score ≥ 1/1. For the update, however, analyses also included workers with B-reader ratings of ILO score ≥ 1/0 or higher (*n* = 156, of whom 48 had ILO score ≥ 1/1). Quantitative exposure estimates were based on a JEM informed by more than 8,000 personal and area sampling measurements obtained as early as 1954. Cumulative exposure categories were defined as ≤0.5; >0.5–1.0; >1.0–1.5; >1.5–3.0; >3–4, >4–5; >5–6 and > 6 mg/m^3^-yrs, respectively, and average exposure categories were defined as ≤0.05; >0.05–0.1; >0.1–0.15; >0.15–0.2 and > 0.2 mg/m^3^, respectively. Exposure estimates were not updated primarily because IH measurements over the extended follow-up years verified uniformly negligible to very low exposure concentrations that would not materially change individual cumulative exposure estimates. Cox proportional hazards models evaluated cumulative exposure, average exposure and duration of employment controlling for age, gender and smoking (ever/never/unknown). Strengths of this study include the large sample size, detailed quantitative exposure information, and the medical/chest radiograph surveillance program that allowed identification of new (i.e., incident) silicosis cases. The initial study was limited by a relatively short follow-up period (median follow-up less than 15 years); however, the update added 15 more years of follow-up and included the ILO score 1/0 silicosis cases. The smoking information from medical records was limited to basic categories of ever/never/missing, which was more relevant to the lung cancer analyses but nevertheless was associated with silicosis (HR = 2.4 95%CI 1.2–4.9) for “ever” compared with “never” smokers.

#### 3.1.3 Foundries

Swedish foundry workers. Lenander-Ramirez et al. (34) followed a cohort of 1,752 workers from 10 iron foundries employed between 1913 and 2005 in Sweden for silicosis mortality. Workers with silicosis were identified either from the National Non-primary Outpatient Register (NPR) or the National Causes of Death Register. Quantitative exposure assessment was based on job categories and informed by both personal and area sampling for total respirable dust and respirable silica dust. Standardized incidence ratios (SIRs) were computed by tertiles of exposure using the general Swedish population as the reference. Only six silicosis deaths were identified, however, limiting the study’s statistical power and ability to address exposure-response patterns.

Chinese foundry workers. Zhang et al. (36) followed through 2008 a cohort of 2,009 workers employed for at least a year during 1980–1996 at an automobile foundry in Shiyan, Hubei province, China, for silicosis defined using China’s National Diagnostic Criteria for Silicosis (with no specific mention of stage or relationship to ILO ratings). Quantitative assessment of silica exposure was based on employment history and air monitoring samples obtained from 1978 to 2008. Multivariate logistic regression was used to evaluate cumulative silica exposure as a continuous variable, adjusting for age, cigarette smoking, and alcohol consumption. A separate logistic regression was performed using only cumulative silica exposure as a continuous variable. Pulmonary tuberculosis was found to influence silicosis risk but was not used in the multivariate models, as only 7 of the 48 silicosis cases had a record of tuberculosis. Limitations include the incomplete information on how silicosis was defined and possible under-ascertainment of tuberculosis.

### 3.2 Silicosis exposure-response results summary

While mean RCS exposure and duration of RCS exposure was reported in most studies, cumulative RCS exposure was estimated and reported in all the studies; therefore, this metric was used for examining exposure-response relationships across studies (Table 2). However, most studies used different RCS exposure category cut points, making it difficult to compare findings directly. Two non-mining/quarrying studies only modeled cumulative exposure as a continuous variable and reported a single relative risk representing the increase in silicosis risk per 1 mg/m^3^-year increase in cumulative exposure (36, 43). Both of these, however, presented very different risk estimates: Cherry et al. estimated a unit odds ratio of 1.37 (1.24–1.53) whereas Zhang et al. (36) estimated an odds ratio of 5.38 (95% CI: 3.82–7.57) per 1 mg-/m^3^-yr, although the estimate from Cherry et al. (43) was more consistent with estimates from other studies for approximately the same cumulative exposure (15, 35, 37, 40). The three non- mining/quarrying studies reported RCS exposure categories with statistically significant increased risk of silicosis, especially in the highest exposure categories of each exposure metric (15, 34, 44). Trends of increasing silicosis risk with increasing cumulative RCS exposure were reported by all six studies that evaluated risks among miners, with strong indications of exposure thresholds—but at very different cumulative (or average intensity) exposure levels. Also, although associations with smoking were not reported in every study, five reported statistically significant associations between smoking and silicosis (15, 36, 38, 43, 44) and one did not (35) (see Supplementary Table S1 for additional details).

**Table 2:** Exposure-response estimates for cumulative silica exposure and silicosis.

Study (First author year)	Exposure categories (mg m^3^-years)	Observed cases	RR* estimate	95% CI
Cherry 1998	per unit cumulative exposure	64 (total)	1.37	1.24–1.53
Hughes 1998	≤1	81 (total)	1.0	referent
	>1–≤3		4.35	1.7–11.1
	>3–≤4		14.88	5.44–41.0
	>4–≤6		25.33	9.9–65.2
	>6–≤8		36.36	12.8–102.4
	>8		43.47	16.7–113.5
Steenland 2001	>0–0.10	1	1	referent
	>0.10–0.51	2	1.22	NR
	>0.51–1.28	4	2.91	NR
	>1.28	7	7.39	NR
Rego 2008	1.21 (0.06–3.23) [mean (range)]	177	1.0	Referent
	4.88 (3.36–6.59)	87	2.81	0.78–10.10
	8.79 (6.75–11.71)	93	10.16	3.3–31.0
	17.78 (11.8–37.93)	83	31.46	10.3–95.5
Zhang 2010	per unit cumulative exposure	48 (total)	5.38	3.82–7.57
Vacek 2011	≤1.04	4	1.00	referent
	1.05–3.64	5	2.02	0.45–9.09
	3.65–6.71	13	8.62	1.86–39.95
	6.72–10.21	17	12.36	2.67–57.2
	>10.21	16	10.55	2.30–48.4
Vacek 2019	≤0.50	7	1.0	referent
	0.51–1.50	7	1.10	0.26–4.64
	1.51–3.00	10	2.06	0.55–7.79
	3.01–5.50	20	12.57	2.08–55.47
	>5.50	23	25.50	5.52–117.82
Wang 2020a (Tungsten mines)	0–0.87	6,760 (total)	1.0	referent
	0.88–2.24		3.90	3.19–4.77
	2.25–5.50		11.24	9.26–13.64
	>5.50		19.50	16.05–23.69
Wang 2020a (Fe/Cu mines)	0–0.87	405 (total)	1.0	referent
	0.88–2.24		1.86	1.48–2.33
	2.25–5.50		4.96	3.51–7.00
	>5.50		9.09	5.57–14.85
Wang 2020a (tin mines)	0–0.87	997 (total)	1.0	referent
	0.88–2.24		3.04	2.27–4.08
	2.25–5.50		5.98	4.47–8.00
	>5.50		6.4	4.50–9.09
Wang 2020a (Pottery factories)	0–0.87	1,215 (total)	1.0	referent
	0.88–2.24		2.88	1.43–5.80
	2.25–5.50		4.88	2.52–9.46
	>5.50		5.76	2.98–11.12
Lenander-Ramirez 2022	≤ 0.14	0	NA	NA
	0.15 to 0.38	0	NA	NA
	0.39+	6	45.87	16.83–99.83
Birk 2025 (ILO ≥ 1/0)	≤0.5	6	1.0	referent
	>0.5–1.0	7	1.6	0.5–5.0
	>1.0–1.5	13	4.8	1.7–13.5
	>1.5–3.0	15	3.6	1.3–10.1
	>3.0–4	7	4.8	1.5–15.5
	>4.0–5.0	4	3.9	1.0–14.8
	>5.0–6.0	9	9.5	3.1–29.3
	>6.0	47	16.3	6.1–43.4
Birk 2025 (ILO ≥ 1/1)	≤0.5	4	1.0	Reference
	>0.5–1.0	2	0.9	0.1–5.5
	>1.0–1.5	2	1.3	0.2–8.1
	>1.5–3.0	2	0.8	0.1–5.1
	>3.0–4	3	3.3	0.6–18.1
	>4.0–5.0	4	6.5	1.3–32.4
	>5.0–6.0	7	10.5	2.4–46.2
	>6.0	24	9.2	2.3–36.8

*Measure of association reported in the studies included RR, SMR, SIR, HR and OR.

**Figure 2:**
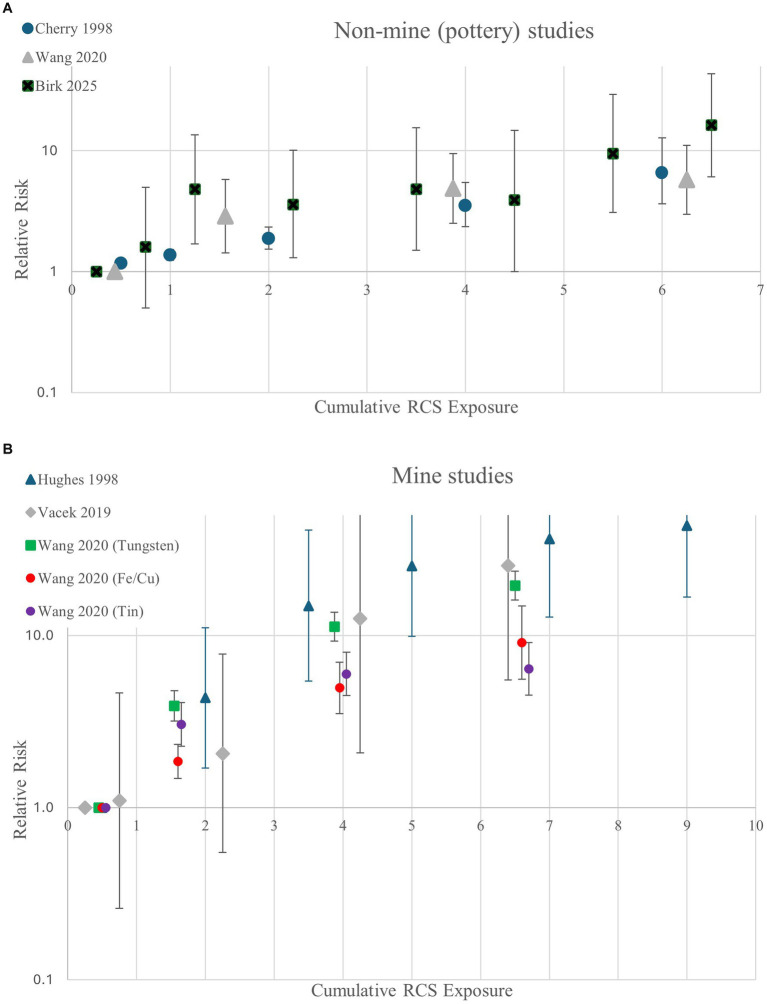
Relative risk estimates for silicosis (≥1/0) by cumulative RCS in **(A)** non-mine (pottery) studies and **(B)** mine studies.

To more closely evaluate the shape of the exposure-response relationship between RCS and the subset of studies defining silicosis as the ILO category of ≥1/0, we plotted the measures of association for the three non-mine studies (all pottery workers) and separately the five results from the three mine studies (see Figures 2A,B). The figures display substantially increased risks above 1 mg-/m^3^-yr in both figures, with much higher risk with higher exposures in the studies of miners.

### 3.3 Lung cancer studies

Table 3 summarizes the 12 studies included for systematic review that addressed lung cancer, were rated in as “medium” or “high” based on the systematic quality evaluation and presented or provided information allowing estimation of exposure-response results. There were eight cohort studies, three nested case–control studies and one pooled analysis of case control studies. Four studies were conducted in the US, and one each in China, Europe/Canada, Finland, Germany, Netherlands, Norway, South Africa and the UK. Two of the studies also were included in Table 1 (39, 44). Five studies focused exclusively on mine or quarry workers, three exclusively on non-mine or quarry settings, and four studies that included workers from both mine/quarry and other occupational settings. The number of lung cancer cases in these studies ranged from 62 cases (45) to about 5,400 cases (18). Studies used a variety of methods to attempt to control for smoking, e.g., four studies used relatively crude categories of ever/never/unknown; five studies estimated pack-years (a measure that combines intensity and duration); and three studies used only indirect (i.e., ecological) adjustment for smoking (18, 19, 46). Only one study presented exposure-response data specifically for never smokers, a subgroup unlikely susceptible to confounding or residual confounding due to cigarette smoking (12).

**Table 3:** Description of studies with exposure-response for silica and risk of lung cancer.

Study (First author year)	Study design	Industry	Country	Earliest employment or year studied	Adjustment variables	Overall # of cases	Exposure assessment method	Outcome assessment method
Hnizdo 1997	Nested case–control	Gold Mining	South Africa	1968	Cigarette smoking (categorical packyears)	78	Work records and average exposure estimates by work category	Death certificates and autopsies
Steenland 2001	Nested case–control	Sand Industry	US	1946	Race, sex, age; indirect adjustment for smoking	75	JEM based on personal and area sampling	Death certificates (ICD-9 code 162)
McDonald 2005 (overlaps with Hughes 2001)	Nested case–control	Sand Industry	USA	1940	Smoking (never, ever and unknown)	105	Company records and mean exposure levels by job	Death certificates (ICD-9 code 162)
Pukkala 2005	Registry and Census based Cohort	Not restricted	Finland	1945	Smoking (indirect), Asbestos, age, calendar period of follow-up, social class, other occupational carcinogens	~5,400	Finnish JEM and reported census occupation	Cancer registry
Preller 2010	Cohort	Multiple Industries	Netherlands	1986	Smoking (current (Y/N), average # smoked, years), alcohol intake, fruits/vegetable intake, family history of lung cancer, age	1,667	Finnish JEM for self-reported lifetime job histories	Cancer registry
Bugge 2012	Cohort	Silicon carbide (SiC)	Norway	1913	Smoking (ever/never/unknown), asbestos, lag time of 20 years; age (3 categories)	62	JEM based on personal sampling	Cancer registry
Cherry 2013 (overlaps with Cherry 1998)	Cohort	Pottery factories	UK	1931	Smoking (current/former/unknown), age, duration of employment	243	Estimated duration and exposure levels by type of work	Cause of death from office of national statistics
Graber 2014	Cohort	Coal Mining	US	1969	Age at study entry, race, coal-rank region, year of birth, smoking status (ever, former, never), smoking pack years and body mass index	568	Estimated using MSHA data and job groupings	Cause of death from National Death Index
Gallagher 2015	Cohort	Diatomaceous earth mining and processing	US	1942	Age at entry, calendar year at entry, ethnicity; Asbestos (supp. Tables); Smoking (indirect)	113	Job category estimates based on air monitoring and relative rankings	Cause of death from National Death Index
Ge 2020	Pooled case–control	Multiple Industries	European countries and Canada	1960	Smoking (packyears); smoking cessation (current smoker; 4 former categories; never), list A jobs, age	2,882	SYN-JEM for self-reported occupational history	Cases from IARC SYNERGY Project
Wang 2020b (overlaps with Lai 2018; Chen 2012; Cocco 2001)	Cohort study	Metal mines and pottery factories	China	1960	Smoking (packyears), sex, year at hire, age at hire, type of facility (tungsten mine, iron and/or copper mine, tin mine, and pottery factory)	917	JEM based on area sampling	Mortality from various sources such as hospital records, employment registers, death certificates, oral reports from participants’ relatives (ICD-10-CM code C33-C34)
Birk 2025	Cohort	Porcelain manufacturing	Germany	1938	Smoking (ever/never/unknown), age, sex, duration of employment	284	JEM based on personal and area sampling	Death certificates (ICD-10)

Studies meeting at least minimal standardized quality assessment standards are summarized below, presented by industrial setting.

#### 3.3.1 Mining and quarrying

South African gold miners. Hnizdo et al. (47) conducted a case–control study nested in a cohort of South African gold miners (*n* = 2,240) that previously generated an excess of lung cancer deaths (48). The 78 lung cancer cases were identified from death certificates and autopsies between 1970 and the end of 1986. Cases were matched to five controls based on year of birth and year of death of the case. Quantitative silica exposure assessment was based on employment records and average exposure estimates for nine occupational categories. Smoking information was acquired via questionnaire during medical examinations conducted from1968 through 1972. Conditional logistic regression models included cigarette consumption (pack years), cumulative silica dust exposure lagged 20 years, number of years worked in dusty occupations lagged 20 years, and silicosis diagnosis. Overall, the study was reasonably strong with a moderate number of cases and detailed smoking information.

US coal miners. Graber et al. (49) evaluated lung cancer mortality among 9,033 coal miners from 31 US mines enrolled during 1969 and 1971. Information on smoking history, work history, demographics and respiratory symptoms were collected via questionnaire at enrollment. Quantitative assessment of silica exposure was constructed through estimates from the Mine Safety and Health Administration compliance data from 1982 to 2002 combined with job categories. Based on searches of the National Death Index, 568 lung cancers were identified through 2007. Cox proportional hazards models evaluated exposure-response relationships of coal mine dust and respirable silica dust with lung cancer, adjusting for age at study entry, race, coal-rank region, year of birth, smoking status (ever, former, never), pack years of smoking at enrollment and body mass index. The study was based on a large number of lung cancer deaths and had adequate smoking and silica exposure information, although exposure history after enrollment was not included.

US industrial sand workers. Steenland and Sanderson (39) conducted a nested case–control study of the 75 lung cancer deaths among workers employed more than 6 months and 100 controls randomly chosen for each case matched on race, sex, and date of birth within 5 years from a cohort of 4,626 industrial sand workers (summarized above in the silicosis section). Logistic regression models evaluated quartiles of exposure (i.e., 0–0.18; >0.18–0.59; >0.59–1.23; and > 1.23 mg/m^3^-yrs, respectively) both unlagged and lagged 15 years. Individual smoking information was not available; however, a sample of 346 men (7.5%) aged 25–64 employed at 4 plants during 1978–1989 was surveyed to obtain smoking histories. Categorical analyses of smoking (never/former/current) and exposure indicated that smokers did not have a higher cumulative dose of silica. An Axelson-type indirect adjustment also was performed, and the effects were minimal (i.e., 10% or less with vs. without adjustment). The cohort of sand industry workers represented a study group unlikely to have co-exposure to other hazards such as radon and diesel fumes; however, the lack of individual smoking information—and the inference that the potential for confounding by smoking was not large based on a relatively small and recent sample of the cohort may be a limitation. Nevertheless, the analysis of the reasonably large group of sung cancers among never-smokers provides evidence unlikely to be greatly affected by confounding by smoking.

McDonald et al. (41) conducted an update of mortality through 2000 for a cohort of 2,452 workers employed for at least 3 years at one of eight US sand-producing plants. Prior publications had noted an elevated lung cancer risk in this cohort (30, 31, 33). A nested case–control analysis was performed for 105 lung cancer deaths with detailed job history and controls matched on plant, birth year within 5 years and date of first hire within 5 years. Quantitative silica exposure estimates were obtained by linking company employment records to mean job exposure levels adjusted for frequency of respirator use. Conditional logistic regression evaluated quartiles of cumulative exposure (i.e., ≤300; >300- ≤ 1,100; >1,100- ≤ 3,300 and > 3,300 μg/m^3^-yrs, respectively) lagged 15 years and using the lowest exposure group as the referent. Smoking information (never, ever, unknown) obtained from employment records was included in the models. Overall, the study had an adequate sample size and reasonable quantitative silica exposure estimates.

US diatomaceous earth workers. Gallagher et al. (46) updated mortality through 2011 of the cohort of 2,342 men employed since 1942 at a diatomaceous earth plant in California (US). This cohort was summarized above in the silicosis section (32, 38) and additional lung cancer results were reported in Checkoway et al. (25). Quantitative respirable silica exposure was based on job category estimates from air monitoring and relative rankings of the jobs. The 113 observed lung cancer deaths identified via the National Death Index (NDI) was close to the number expected and SMR = 1.03, 95% CI: 0.85–1.23. Cox proportional hazards models with 0, 10 and 15-year lags, respectively, were developed to evaluate cumulative silica exposure and lung cancer mortality adjusting for age at entry, calendar year at entry, and ethnicity. A variable for asbestos exposure was added and results are summarized in the Supplementary materials. The authors addressed smoking status (ever/never available for 50% of the cohort) by applying the Axelson indirect adjustment method. The updated results reported by Gallagher et al. (46) were similar to those presented in Checkoway et al. (25), indicating the previously reported moderate excess risk of lung cancer in high cristobalite exposure categories remained consistent. Overall, this cohort is informative and has adequate exposure information, but not having individual smoking information hampers the interpretation of results.

Chinese metal miners and pottery workers. Wang et al. (16) reported on a cohort of 44,708 workers employed in 20 tungsten, tin or iron/copper mines and 9 pottery factories in central and southern China for at least one between 1960 and 1974. The study population was the same as reported in Wang et al. (15) but with different exclusions. Quantitative silica exposure assessment for this study was the same as reported in Wang et al. (15). Several prior publications of this cohort evaluated silica in relation to lung cancer mortality risk (13, 26, 29). Detailed information on cigarette smoking was collected via questionnaires in 1986, 1995 and 2004. Information on mortality was obtained from hospital records, employment registers, death certificates, and oral reports from participants’ relatives. Cox proportional hazard models estimated quantitative exposure-responses between cumulative silica exposure and lung cancer (*n* = 917) adjusting for smoking (pack years), sex, year of hire, age at hire, and type of facility. The study is notable for its size, length of follow-up, and detailed information on smoking history and dust exposure, although nearly 40% of the cohort was excluded due to missing information on smoking and/or work history. However, results were not provided separately for metal miners and pottery workers as in Wang et al. (15).

#### 3.3.2 Ceramics and porcelain workers

British pottery workers. As summarized above, the mortality of a cohort of pottery workers in Stoke-on-Trent England was reported in two publications (42, 43), the second updated exposure-response analysis for lung cancer (42). Over the entire cohort period (1985–2008), 243 lung cancer deaths were identified from the office of national statistics, compared with 211.72 expected based on rates for Stoke-on-Trent. Details of the methods and adjustment factors for the exposure-response analysis for silica exposure were not reported, although limited smoking status (i.e., current, former or unknown) information was available. Quantitative silica exposure assessment was not updated. This study had adequate silica exposure information; however, few details were provided on the exposure-response analysis for lung cancer.

German porcelain workers. Birk et al., (44), updating Mundt et al. (14), identified 194 lung cancer deaths among men and 90 among women, based on coded cause of death on the death certificates. No excess of lung cancer deaths was observed (SMR = 0.90, 95% CI 0.78–1.04 among men or among women SMR = 0.98 95% CI 0.80–1.21) based on state (i.e., Bavarian) reference rates. Cox proportional hazards models evaluated cumulative exposure over eight categories (using the lowest category as the referent), average exposure and duration controlling for age, gender and smoking (ever/never). Duration of employment also was unrelated to lung cancer risk. Strengths of this study include the large sample size, long follow-up and detailed quantitative exposure information. Although smoking history information was limited to crude categories of ever/never/unknown, HR = 17.9 (95% CI 7.3–43.7) and HR = 6.1 (95% CI 3.4–10.9) for ever versus never smoking and lung cancer for men and women, respectively.

#### 3.3.3 Silicon carbide production

Norwegian silicon carbide workers. Bugge et al. (45) evaluated cumulative respirable silica exposure (quartz), as well as exposure to cristobalite, silicon carbide dust and silicon carbide fibers, and risk of lung cancer in a cohort of 1,687 workers employed for three or more years during 1913–2003 in the Norwegian silicon carbide industry. Lung cancers (*n* = 62) occurring during 1953–2008 were identified from the Norwegian Cancer Registry. Quantitative assessment of silica and other exposures were derived using employment records and a JEM. Smoking information (ever, never or unknown) was obtained from plant occupational health services departments. Exposure-response analyses were limited to SIRs by tertiles of cumulative exposure, unlagged and lagged 20 years. Analyses were constructed by department and by cumulative exposure to all factors except asbestos. Quartz was not addressed in the multivariate analyses. Although the study has several strengths, its limited ability to differentiate risks associated with quartz from those due to cristobalite exposure (a different form of crystalline silica) as well as silicon carbide dusts, fibers and whiskers. Smoking history was available on an individual level, but in broad categories of ever, never and unknown.

#### 3.3.4 Population-based (not industry specific)

Pukkala et al. (18) conducted a census-based registry study of the Finnish population born in 1906–1945 who participated in the 1970 national census and were employed in various industries. Quantitative silica exposure estimates for specified calendar periods, occupations, and exposure agents were based on the Finish job exposure matrix (FINJEM). A total of 43,433 lung cancer cases was identified 5,521of which were analyzed in the “preferred model,” a Poisson regression analysis using a lag time of 20 years and adjustment for age, period, social class, smoking and asbestos with the unexposed group as the comparison. Smoking prevalence was obtained from FINJEM by occupation but was unavailable at the individual level. The strengths of this study include the very large number of incident lung cancer cases ascertained from a cancer registry and likely exposed to silica. Limitations of this study include the approximation of exposure based upon census employment data and the limited indirect information on smoking prevalence by occupation rather than at the individual level.

Preller et al. (50) evaluated 1,167 lung cancer cases identified over 11.3 years of follow-up of a population-based cohort of 58,279 men aged 55–69 and participating in the Netherlands Cohort Study. Employment information was self-reported and quantitative assessment of silica exposure was estimated for 210 cases with likely silica exposure based on FINJEM. Cox proportional hazards models evaluated exposure-response relationships adjusting for smoking (current: yes/no, average number of cigarettes, and years smoked), alcohol intake, fruit/vegetable intake, family history of lung cancer, and age. Although the study has adequate size and detailed smoking history, limitations include the use of self-reported occupational history as a basis for quantitative estimates of silica exposure.

As part of the IARC SYNERGY project, Ge et al. (12) evaluated silica exposure and lung cancer in a pooled analysis of 14 hospital- and population-based case–control studies conducted in Europe and Canada. Lung cancer cases were identified from registry or hospital records. Of the 16,901 lung cancer cases, 4,923 were likely to have had occupational silica exposure. Quantitative silica exposure assessment was based on self-reported occupational history and the Synergy project JEM (SYN-JEM). Silica concentrations for years prior to 1960 were assumed to be the same as those in 1960. Unconditional logistic regression models were developed adjusting for study, age group, sex, smoking (pack-years, time since quitting) and “list A” jobs, i.e., those that had “known occupational lung cancer risks (e.g., welders, long-distance truck drivers, or boiler operators).” Results stratified by smoking status (i.e., never, former, current) also were presented. The study is notable for the large number of lung cancer cases with potential silica exposure, including an adequate number among never smokers so that the association between silica exposure and lung cancer could be assessed reasonably free of confounding and residual confounding due to smoking. Possible limitations include the lack of details on case ascertainment and reliance on self-reported occupational history.

### 3.4 Lung cancer exposure-response results summary

As with the silicosis studies, cumulative RCS exposure was estimated and reported in all lung cancer studies. Mean RCS exposure and duration of RCS exposure were not consistently reported across studies; therefore, cumulative exposure served as our primary metric for exploring exposure-response relationships (see Supplementary Table S2). Exposure-response estimates for the 12 studies in Table 3 were mixed and are summarized in Table 4. Four studies reported no statistically significantly increased relative risk of lung cancer with any level of cumulative RCS exposure (42, 44, 49, 50). Two studies reported statistically significant trends (39, 41) without reporting any statistically significant elevated relative risk estimates for individual exposure categories. Three studies reported statistically significant increased risk of lung cancer only for the high or highest cumulative RCS exposure categories (18, 46, 47). Three studies reported increased risks of lung cancer across all exposure categories (12, 16, 45). As smoking was the largest risk factor for lung cancer with relative risk estimates ranging from 2.7 to 20.9 (see Supplementary Table S2), raising the possibility of residual confounding in studies lacking individual-level smoking information. Additional review of the 12 lung cancer studies found that residual confounding generally was not explicitly discussed. However, one study presented an exposure-response analysis for silica and lung cancer (*n* = 248) specifically limited to never-smokers with substantial silica exposure (12). The odds ratio was not increased until the highest RCS exposure category; however, because it is open-ended (≥ 2.4 mg/m^3^-yrs), it is not possible to estimate with any precision the range or typical exposure level in this group; therefore, the cumulative exposure level (i.e., threshold) at which lung cancer risk substantively increases cannot be quantified precisely (12).

**Table 4:** Exposure-response estimates for cumulative silica exposure and lung cancer.

Study (First author year)	Exposure (mg x years/m^3^)	Observed Cases	RR* Estimate	95% CI
Hnizdo 1997 (Model 1)	2.7–4.3	464 (total)	1.83	0.8–4.10
	4.4–6.3	464 (total)	1.85	0.8–4.30
	>6.3	464 (total)	3.19	1.3–7.60
Steenland 2001	0–0.18	20	1.00	reference
	>0.18–0.59	21	1.35	0.72–2.54
	>0.59–1.23	18	1.63	0.83–3.18
	>1.23	16	2.00	1.00–4.01
McDonald 2005	≤0.3	13	1.00	NR
	>0.3–≤1.1	17	0.94	NR
	>1.1– ≤ 3.3	38	2.24	NR
	>3.3	37	2.66	NR
Pukkala 2005	≤0.9	3,115	1.05	1.00–1.10
	1.0–9.9	2,277	0.97	0.91–1.03
	≥10	129	1.42	1.20–1.70
Preller 2010	>0 to <3	148	0.95	0.73–1.25
	≥3	62	1.47	0.93–2.33
Bugge 2012	0–0.026	29	1.4	1.0–2.0
	0.026–0.077	15	1.8	1.1–3.0
	0.077–2.3	18	2	1.3–3.3
Cherry 2013 (mean exposure concentration)	<0.1	117 (total)	1.00	Referent
	0.1– < 0.15	117 (total)	1.07	0.65–1.74
	0.15– < 0.2	117 (total)	0.76	0.43–1.32
	≥0.2	117 (total)	0.96	0.58–1.60
Graber 2014	<2.22	568 (total)	1.0	referent
	2.22–3.30	568 (total)	1.08	0.85–1.37
	3.31–4.12	568 (total)	1.20	0.95–1.52
	≥4.13	568 (total)	1.17	0.92–1.50
Gallagher 2015	<0.4	29	1.00	referent
	0.4–<0.9	17	1.38	0.76–2.52
	1.0–<2.6	27	1.24	0.73–2.10
	2.6– < 5.6	20	1.98	1.11–3.54
	>5.6	20	2.36	1.25–4.46
Ge 2020 (never smokers)	Never	1,121	1.00	referent
	>0–0.39	60	1.17	0.85–1.57
	0.4–1.09	59	1.07	0.78–1.43
	1.1–2.39	60	1.02	0.75–1.36
	≥2.4	69	1.4	1.03–1.86
Ge 2020 (former smokers)	Never	3,696	1.0	referent
	>0–0.39	366	1.07	0.92–1.25
	0.4–1.09	433	1.37	1.18–1.59
	1.1–2.39	441	1.35	1.16–1.57
	≥2.4	496	1.47	1.27–1.70
Ge 2020 (current smokers)	Never	7,161	1.00	referent
	>0–0.39	687	1.19	1.03–1.39
	0.4–1.09	729	1.33	1.15–1.55
	1.1–2.39	730	1.29	1.11–1.50
	≥2.4	793	1.39	1.20–1.62
Wang 2020b**	0 to 1.056	917 (total)	1.32	1.07–1.62
	1.057–3.925	917 (total)	1.51	1.25–1.83
	>3.925	917 (total)	1.52	1.24–1.87
Birk 2025 (men)	≤0.5	45	1.0	reference
	>0.5–1.0	25	0.8	0.5–1.3
	>1.0–1.5	18	0.8	0.5–1.4
	>1.5–3.0	35	0.9	0.6–1.5
	>3–4	13	1.2	0.7–2.3
	>4–5	10	1.2	0.6–2.4
	>5–6	14	1.5	0.8–2.8
	>6	34	1.0	0.6–1.6
Birk 2025 (women)	≤0.5	27	1.0	Reference
	>0.5–1.0	22	1.0	0.6–1.8
	>1.0–1.5	7	0.5	0.2–1.2
	>1.5–3.0	16	0.7	0.4–1.4
	>3–4	8	1.1	0.5–2.6
	>4–5	4	0.8	0.3–2.2
	>5–6	2	0.5	0.1–2.0
	>6	4	0.6	0.2–1.7

*Measure of association reported in the studies included RR, SIR, HR and OR. **results presented only for miners and pottery workers combined in (16).

**Figure 3:**
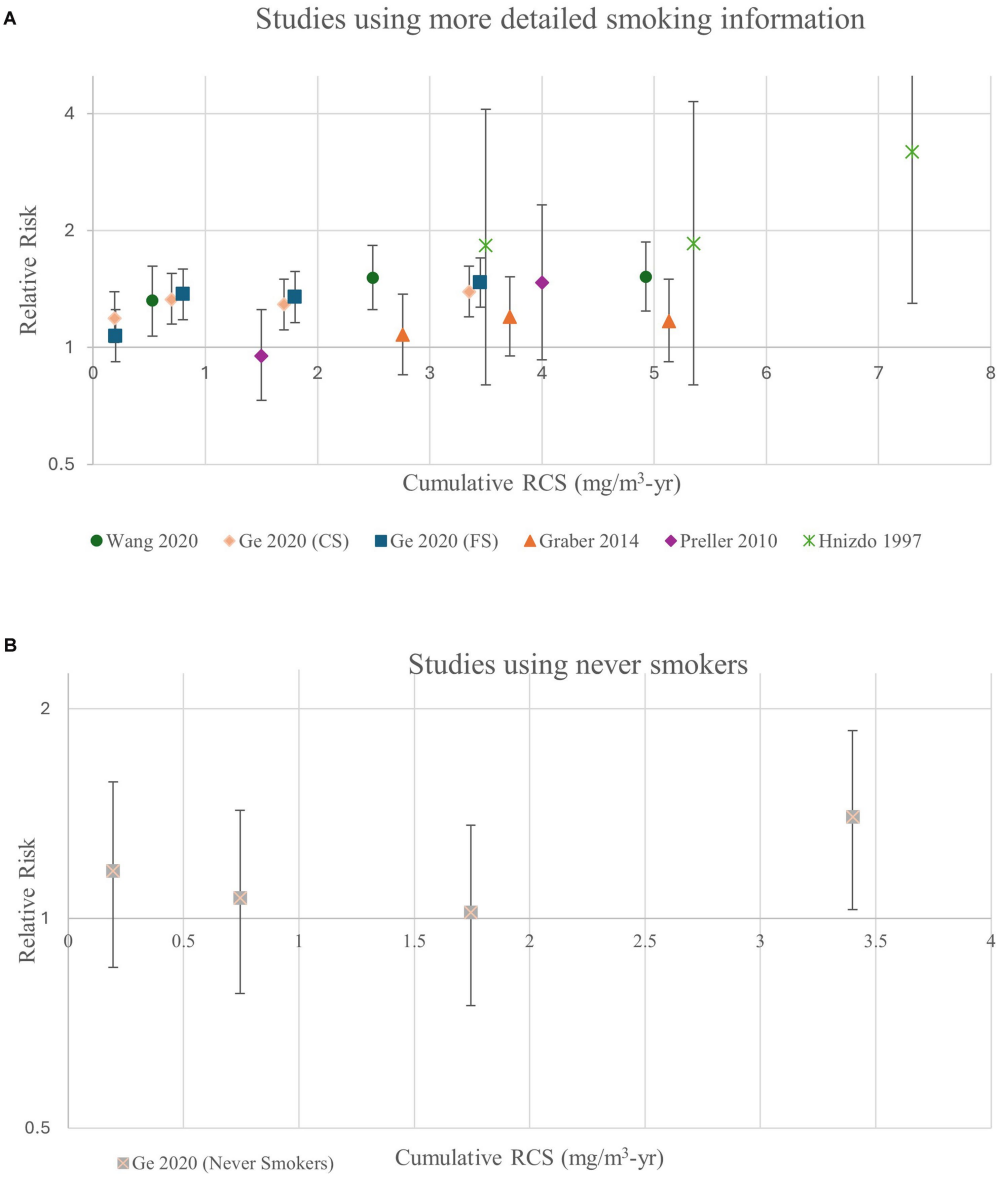
Relative risk estimates for lung cancer by cumulative RCS in **(A)** studies using more detailed smoking information (duration/intensity) and **(B)** never smokers.

To more closely evaluate the shape of the exposure-response relationship between RCS and the subset of lung cancer studies using more detailed smoking information including estimates of intensity and/or duration, we plotted the measures of association for the five studies (all pottery workers) with detailed control of smoking and separately the single results from the one study on never smokers (see Figures 3A,B). The figures display risks that hover around baseline except at the very highest exposure categories.

## 4 Discussion

The body of epidemiological studies on RCS and risk of silicosis and lung cancer is relatively large and complex; however, the number of studies of at least moderate quality sufficient to evaluate quantitative exposure-responses and exposure thresholds for these diseases is relatively small. Specifically, of the hundreds of publications identified using standard search methods and three publication indexing databases (i.e., PubMed, Web of Science and Google Scholar) screened, only 31 studies initially were determined to provide at least semi-quantitatively estimated RCS exposures and estimated relative risks for silicosis, lung cancer or both, with the additional requirement that those assessing lung cancer risks also take into account potential confounding by tobacco smoking, the most common known cause of lung cancer.

Even among this subset of relevant papers there were some overlapping study groups, largely due to pooling or combining of previous studies for analysis, expanding and/or updating previously defined cohorts, and additional analyses of subsets of previous studies, including case–control analyses nested in previously published cohort studies. Furthermore, an updated or combined study may not have retained all cohort members from the previous study. For example, Lai et al. (13) followed 7,665 iron workers whereas Wang et al. (15, 16) had 4,653 iron and copper workers. This made it difficult if not impossible to isolate the most recently updated, non-overlapping (i.e., independent) study groups for comparison and synthesis of reported results. Generally, however, updated cohorts were favored as they included the most up-to-date results and longest follow-up, possibly providing greater numbers of cases with appropriately long latency.

### 4.1 Silicosis

Systematic review of the most up-to-date and relevant studies of quantitative RCS exposure estimates and silicosis risk revealed additional factors that made it difficult to compare studies directly and therefore synthesize study results. The most important of these was the non-comparable ways historical exposures were quantitatively estimated and exposure categories numerically defined. The variability in the way the referent (and other) exposure groups were defined across studies also directly influenced the quantitative risk estimates generated, further complicating their possible (and precluding direct) comparison. The stability of the referent group—directly related to the number of silicosis cases or deaths observed (where none or nearly none would be expected as silicosis cases or deaths would not be expected among groups not substantially exposed to RCS)—largely determined the overall variability of most statistical results. Analyses relying on referent groups with zero silicosis cases or deaths would require division by zero, which is mathematically undefined. Steenland and Sanderson (39) defined four cumulative exposure categories: >0–0.10; >0.10–0.51; >0.51–1.28; and > 1.28 mg/m^3^-yrs, respectively, the first of which served as the reference group, despite only having one silicosis death, resulting in unstable relative risk estimates for all higher exposure categories. Had the authors combined the lowest two exposure categories, there would have been three cases in the reference group defined as >0–0.51 mg/m^3^-yrs and comparable with several other studies, with somewhat increased precision of the risk estimates. Many studies redefined reference groups to include some (generally small) numbers of silicosis cases, even if such cases may not reflect actual risks associated with those exposure levels, attenuation relative risk indicators in higher exposure groups, and theoretically obscuring exposure thresholds.

As a consequence of defining reference groups based on the occurrence of silicosis in low exposure groups, exposure categories across studies varied widely. For some studies, the lowest exposure category—and reference group—included individuals estimated to have had cumulative exposures that subsumed many and sometimes all exposure categories in other studies. For example, Birk et al. (44)and Vacek et al. (40) at least initially defined reference groups as those with ≤0.5 mg/m^3^-years. However, because very few silicosis cases were identified among porcelain workers with cumulative exposure estimated to be ≤3.0 mg/m^3^-years, and because initially estimated HRs for each of the next three cumulative exposure categories were similar to (or less than) the referent, Birk et al. (44) redefined the referent group to include all workers with ≤3.0 mg/m^3^-years and further stratified those with >3 mg/m^3^-years (with a large majority of cases) into categories defined as >3–4; >4–5; >5–6; and > 6. Similarly, as no silicosis case occurred among individuals in the first exposure quintile, but four were observed in the second, Rego et al. (35) combined the first two quintiles to form the referent group with individual exposures ranging from 0.06 to 3.23 mg/m^3^-years.

Additionally, nearly all studies defined the highest exposure category as all workers with some numerical lower bound of cumulative exposure but with no upper limit identified, i.e., an open-ended category. Therefore, for example, where the highest cumulative exposure group in Vacek et al. (40) was defined as >5.5 mg/m^3^-years, it is not easily determined whether those most highly exposed had exposure estimates closer to or much higher than 5.5. Lenander-Ramirez et al. (34) defined three exposure categories; however, no silicosis cases were observed in either of the first two categories, therefore all (*n* = 6) cases fell into the open-ended highest exposure category of 0.39+ mg/m3-years, providing no basis for refining that estimate (in part due to the very small number of silicosis cases). Based on all studies reviewed, exposure thresholds appear to range from as low as 0.39 mg/m^3^-years, which is several-fold less than reported in three other studies suggesting cumulative exposure thresholds at or above 3.0 mg/m^3^-years, the highest of which (35) was above 6.75 mg/m^3^-years. Birk et al. (44) estimated a threshold of >3.0 mg/m^3^-years based on silicosis defined as ILO ≥1/1 but a much lower threshold of >1.0 mg/m^3^-years when silicosis was defined as ILO ≥ 1.0, suggesting that the 1/0 rating may be more sensitive, as would be expected, but also more susceptible to false-positive scoring.

Table 5 summarizes the lowest cumulative exposure category from each study that reports a statistically significantly increased relative risk estimate for silicosis risk or mortality, presented from lowest to highest. Although this approach has limitations (e.g., sample size limitations and chosen exposure category cut points), it provides one transparent method to portray the range of reported threshold effects.

**Table 5:** Summary of apparent cumulative exposure thresholds for silicosis risk or mortality by study.

Study	Exposure category	n	RR estimate	Comments
Cherry 1998	per 1 mg/m^3^-yr	64 total ILO > 1/0	1.37 (95% CI 1.24–1.53)	Per unit cumulative exposure
Hughes 1998	>1–≤3	81 total	4.35 (95% CI 1.7–11.1)	
Rego 2008	6.75–11.71	93	10.16 (95% CI 3.3–31.0)	
Zhang 2010	per 1 mg/m^3^-yr	48 total	5.38 (95% CI 3.82–7.57)	Per unit cumulative exposure
Vacek 2011	3.65–6.71	13	8.62 (95% CI 1.86–39.95)	
Vacek 2019	3.01–5.50	20	12.57 (95% CI 2.08–55.47)	
Wang 2020a	0.88–2.24	6,760 total	3.90 (95% CI 3.19–4.77)	Tungsten mines
	0.88–2.24	1,215 total	2.88 (95% CI 1.43–5.80)	Pottery factories
	0.88–2.24	405 total	1.86 (95% CI 1.48–2.33)	Fe/Cu mines
	0.88–2.24	997 total	3.04 (95% CI 2.27–4.08)	Tin mines
Lenander-Ramirez 2022	0.39+	6	45.87 (95% CI 16.83–99.83)	
Birk 2025	>1.0 >3.0	15 of 156 38 of 48	3.6 (95% CI 1.5–8.5) 7.4 (95% CI 2.0–27.5)	ILO ≥ 1/0 ILO ≥ 1/1

Although Steenland and Sanderson (39) presents SMRs and RRs by exposure category, and the header of the table where relative risk estimates are presented indicates that standard deviations are reported but were not; therefore, it is not possible to determine which if any of the RR estimates reflects statistically significant increased relative risks or threshold estimates.

Two studies modeled exposure-response and reported a single per-unit-of-exposure relative risk estimate, reporting very different unit relative risk estimates (36, 43). Although it is not possible to identify any exposure threshold based on modeled results using a single parameter (which characterizes a slope across all exposure categories probably including high exposure groups with very high relative risks), we present the estimated HRs for a 1 mg/m^3^-year unit increase. This should be interpreted cautiously, as although the unit risks (similar to the slope of a monotonic exposure-response function) were statistically significant, they likely are leveraged by high risks at high exposure categories but insensitive to the exposure level at which risk increased from background to something significantly greater. In other words, modeled unit risks are averages across all exposure levels and unlikely accurately reflect the unit risk at any specific point on the exposure distribution.

Since cumulative exposure to RCS is a function of intensity and duration, it is likely correlated to some extent with duration of employment and age, making disentangling of the separate effects of these problematic. Except in Zhang et al. (36) (see Supplementary Table S1), in none of the studies included in the review was employment duration strongly associated with increased risk of silicosis. However, Zhang et al. (36) reported an average age at onset of silicosis of 47.83 years with average length of employment of 25.94 years. Similarly, Wang et al. (15) reported an average age at onset of silicosis of 45.3 years with net years in dusty jobs of 18.6 years. While our review did not specifically evaluate the independent effect of age on exposure, these results are consistent with the hypothesis that higher exposure to RCS at younger ages (i.e., less than 35 years old) may be more strongly related to silicosis (51). This increased risk for those first exposed at younger ages also could be the result of new employees being assigned to jobs with higher RCS exposures.

Interestingly, and in contrast with some studies indicating little or no association between cigarette smoking and silicosis risk, Birk et al. (44) reported roughly a doubling of silicosis risk among both male and female smokers, which was statistically significant among men. Zhang et al. (36) reported an OR of 4.79 (95% CI 2.24–10.27) for cigarette smoking and the “Diagnostic Criteria of Pneumoconioses,” raising the question of whether this surrogate for silicosis was overly sensitive to smoking-related effects and less specific to silicosis. However, Wang et al. (15) reported that the joint effect of smoking and RCS was at least multiplicative and primarily driven by those in their high exposure category (> 1.81 mg/m^3^-years). While smoking is a strong risk factor for COPD and emphysema, it has also been postulated to contribute to silicosis in silica-exposed workers by exacerbating oxidative stress, inflammation and fibrosis in lung tissues (52, 53). Nevertheless, few of the epidemiological studies reviewed were able to directly quantify—or even address—possible joint effects of smoking and RCS exposure. The few that provided some suggestion of non-independence were unable to clarify whether or not more highly exposed workers also were more likely to have been heavier smokers.

Ultimately, and independently of smoking, most studies (with some exceptions) with sufficient numbers of silicosis cases or deaths and multiple exposure categories over a broad range of RCS exposure values, reported statistically significantly increased relative risk estimates for workers in exposure categories at and above 1.0 mg/m^3^-years—with some threshold estimates at or above 3.0 mg/m^3^-years.

For all studies, however, individual historical RCS exposure estimates carry substantial uncertainties, and the general trends and thresholds observed may reflect the limits of the exposure estimates and the analytical methods applied. Specifically, no study had individual exposure measurements for each worker over their working history; therefore, it is probable that all exposure estimates were subject to considerable misclassification—at least non-differential and possibly systematic misclassification in some settings (e.g., exposure measurements might be more likely performed during upset conditions that may not be representative of levels under usual use or production conditions). Where increased risks at high levels are demonstrated, as with all studies of RCS and silicosis risk, linear models tend to underestimate quantitative relative risks at high exposure levels, and overestimate relative risks at lower exposures, especially including those below any exposure threshold for risk.

Additionally, silicosis diagnostic acuity varied across studies, and likely played some role, as the specific definition of silicosis and the means by which silicosis cases (or deaths) were classified varied across studies and presumably over time as imaging technologies improved. In studies based on data from mandatory medical surveillance programs, e.g., Mundt et al. (14), updated by Birk et al. (44), case ascertainment likely was more complete and representative. In fact, upon re-reading of all chest radiographs for individuals with any single film scored as ILO score 1/0 or higher, Mundt et al. (14) reported strong over-reading (i.e., highly conservative scoring) in the official reading results intended for worker compensation purposes, with no examples of under-reading. This clear potential reader bias likely was eliminated by the systematic re-reading of x-rays following strict ILO/B-reading standards.

### 4.2 Lung cancer

Lung cancer background risks identified in most cohorts following men over the second half of the last and the first decades of this century are based on relatively large numbers of lung cancer cases or deaths in the non- and least-silica-exposed groups, as background rates of lung cancers unrelated to RCS historical have been relatively high. It is well known that lung cancer rates are driven by cigarette smoking history (primarily duration, but also intensity) not only among workers, but also in the general population. Therefore, most any referent group of appropriate size, sex and age structure will be expected to include adequate numbers of lung cancer cases or deaths to derive reasonably stable reference rates.

However, because of the very strong association between cigarette smoking and lung cancer risk, lung cancer studies bear a greater burden of measuring and controlling for the effects of confounding due to cigarette smoking. Simply, if occupational groups highly exposed to RCS smoked more cigarettes, or over more years, relative to whatever group is used as the referent, substantial confounding is possible. Even where some effort to control for confounding by smoking is possible, the threat of residual confounding remains, especially for small effects or where relative risks appear not to be associated with the level of exposure (i.e., no exposure-response). In identifying the set of studies selected for this review, those in which it was possible to at least partially evaluate and/or control for smoking at the individual level were considered stronger and preferentially included. Nevertheless, none of the studies selected was able to obtain detailed individual smoking histories that included duration and intensity indicators, and generally were left stratifying by or controlling for smoking based on smoking information obtained from medical and other records. Even using such relatively crude surrogates, however, Birk et al. (44) reported an HR = 17.0 (95% CI 4.1–69.8) and HR = 4.8 (95% CI 1.1–20.4) for men who were “ever” and “unknown” smokers, respectively, relative to the risk among “never” smokers, indicating that the bulk of the lung cancer risk is driven by smoking, and that even larger HRs likely would have been seen with more precise characterization of smoking history. Therefore, residual confounding by smoking could bias relative risk estimates away from the null (i.e., overestimate), or generate a spurious association.

In contrast with the strong and consistently increased risk of silicosis among groups at least moderately to highly exposed in all studies of RCS and risk of silicosis, results from the studies of RCS and lung cancer (some studies evaluated both) generally are less striking. In two large studies, statistically significantly increased relative risks were reported among miners with >6.3 mg/m^3^-years estimated cumulative exposure (47) and ≥ 10 mg/m^3^-years (18). These exposure groups are well above the observed thresholds seen for silicosis risk. Gallagher et al. (46) reported HRs of 1.98 (95% CI 1.11–2.54) and 2.36 (95% CI 1.25–4.46) in the two highest exposure groups, i.e., 2.6–5.6 and > 5.6 mg/m^3^-years, respectively.

On the other hand, Bugge et al. (45)—a study of silicon carbide workers—reported statistically significant increased SIRs for exposure groups with very low cumulative exposure estimates, i.e., those exceeding 0.026 mg/m^3^-years. Smoking reportedly was statistically controlled, but there was only one lung cancer observed among never smokers, greatly limiting the ability to fully and precisely control for confounding. Wang et al. (15, 16) also reported modest but statistically significant HRs of 1.32–1.52 across the three exposure categories of <1.056; 1.057–3.925; and > 3.925 mg/m^3^-yrs, respectively. The lack of increasing risk across large differences in exposure suggests that the weakly increased HRs might reflect residual confounding due to cigarette smoking—although smoking histories were obtained via participant questionnaires—or some other bias.

Ge et al. (12) was the largest and one of the best-quality studies reviewed, also reporting several weakly increased but statistically significant ORs, especially for analysis stratified by current (ORs of 1.19, 1.33, 1.29, and 1.39) and by former (ORs of 1.07 (ns); 1.37; 1.35; and 1.47) smoking across four exposure groups of <0.39; 0.4–1.09; 1.1–2.39 and ≥ 2.4 mg/m^3^-yrs, respectively, with slight trends noted. They highlighted that these associations were stronger among small cell and squamous cell lung cancers and weaker for adenocarcinoma of the lung—but these patterns also are true for cigarette smoking and might reflect residual confounding. This could help explain why Ge et al. (12) reported a statistically significant multiplicative interaction for lung cancer overall with ever smoking and ever silica exposure, although not by specific histologic type of lung cancer. The lack of clearly increasing risk across groups with more than fivefold differences in cumulative exposure also might reflect exposure misclassification, which can attenuate the slope of exposure-responses; however, although exposure assessment in this study was considered one of its strengths, it relied on *post hoc* expert assessment and assignment, and not exposure measurements.

Perhaps more informative, Ge et al. (12) presented separate statistical results for the sizeable subset of never smokers substantially exposed to RCS, with 248 observed lung cancer deaths. Among all never-smokers, comparing ever- versus never silica-exposed workers generated an OR = 1.02 (95% CI 0.87–1.19). suggesting no overall increased risk associated with silica exposure. Categorical analyses across the same exposure categories generated HRs of 1.17, 1.07, 1.02 and 1.4, respectively, and only the HR for those in the highest exposure group (≥2.4 mg/m^3^-yrs, open ended) was statistically significantly elevated. Because this category is open-ended, it cannot be determined where above 2.4 mg/m^3^-yrs an exposure threshold might lie (note that in their Figure 1; Ge et al. (12) plot ORs for exposures spanning from zero to 7 mg/m^3^-yrs).

Other studies including Preller et al. (50), Cherry et al. (42), Graber et al. (49), and Birk et al. (44) found no statistically significantly increased risks of lung cancers for any silica exposure category. In fact, Birk et al. (44) reported SMR = 0.90 (95% CI 0.78–1.04, based on 194 observed lung cancer deaths) for men and SMR = 0.98 (95% CI 0.80–1.21, based on 90 observed lung cancer deaths) for women, indicating no increased occurrence of lung cancers among members of this cohort. Analyses using the least exposed group as the referent demonstrated no association with exposure, even among the highest exposed group [OR = 1.0 95% CI 0.6–1.6 for exposure >6.0 mg/m^3^-years, based on 38 lung cancer deaths for men and women combined (44)]. These four studies, therefore, provide little evidence of increased risk of lung cancers or any clear association with RCS exposure. Steenland and Sanderson (39) reported elevated standardized risk ratios for lung cancer, but did not report confidence intervals or statistical tests other than p-trend >0.07 (not statistically significant).

Although mixed, the evidence overall suggests some increased risks in some studies, generally limited to groups with high (i.e., 2 or 3 mg/m^3^-yrs) to very high (>4 mg/m^3^-yrs) cumulative exposures. However, Graber et al. (49) and Birk et al. (44) reported no statistically significant positive associations directly with cumulative RCS exposure, even among the highest exposure group (≥4.13 mg/m^3^-yrs and > 6.0 mg/m^3^-yrs, respectively). IARC Monograph (100F) (54) identified seven meta-analyses of lung cancer risks among individuals diagnosed with silicosis and reported relative risk estimates ranging from 1.69 to 3.27. However, the individual studies included in this SR that evaluated both silicosis and lung cancer likely observed too few silicosis cases to evaluate subsequent lung cancer risk among workers previously diagnosed with silicosis.

These mixed findings likely reflect some combination of increased risk at very high RCS exposure levels and with apparently increased risk at lower exposures due to residual confounding by cigarette smoking.

## 5 Conclusion

Based on the results of the studies classified as of reasonable quality and estimating exposure-responses between occupational RCS and risk of either silicosis or lung cancer, risks of these diseases most clearly are increased at substantial cumulative exposure levels. The evidence is strongest and most consistent for silicosis, with most studies indicating significantly increased risks at or above roughly 3 mg/m^3^-yrs and above and some suggesting lower exposure thresholds around 1.0 mg/m^3^-yrs. Even assuming constant ambient RCS levels for 100 years at 3 μg/m^3^ for a 100-year lifespan would generate a cumulative exposure of 0.3 mg/m^3^-yrs, three times lower than the typical exposure thresholds for silicosis (of 1.0 mg/m^3^-yrs) seen in most of the occupational studies and similar to the lowest threshold estimate observed.

A comparable summary for lung cancer is less straightforward, as results from the occupational epidemiological studies reviewed were heterogeneous, including studies observing no increased risk of lung cancers among the most highly exposed cohort members—levels strongly associated with increased silicosis risk. Although some recent reviews and meta-analyses have reported increased risk of lung cancer at relatively low levels of exposure (20, 55), our evaluation demonstrates that risk of lung cancer may not be associated with exposure levels below those at which silicosis risks is elevated, and that preventing silicosis could prevent silica-related lung cancer. Nevertheless, and assuming that at some exposure level risk of lung cancer is increased, risk assessment methods can be applied to estimate anticipated lung cancer risks at the highest levels of ambient RSC. Unfortunately, by likely confounding and possible residual confounding by cigarette smoking, direct extrapolation from the occupational epidemiological studies to characterize increased risks, if any, at ambient exposure levels may not be valid. Possibly the most informative data were presented by Ge et al. (12), i.e., those isolating risks of lung cancer among never-smokers exposed to RCS, for which a cumulative exposure threshold is indicated somewhere greater than 2.4 mg/m^3^-years.

For both silicosis and lung cancer, one scientific question that will have bearing on regulation and occupational health protections is whether current occupational exposure limits are protective against silicosis, and if so—and assuming lung cancer risks increase at levels above those that give rise to silicosis—what are the exposure thresholds for each? Because of the real potential for confounding and residual confounding by cigarette smoking in most studies published to date, the question more straightforwardly might be addressed for silicosis (although a few studies reported moderate and statistically significant associations between smoking and silicosis, possibly reflecting diagnostic bias). If that threshold reliably can be determined—and it falls below the exposure threshold for lung cancer risk—it may not be necessary to derive the exact exposure thresholds to prevent lung cancer. Nevertheless, some robust studies reporting clear excesses and exposure thresholds for silicosis report no increased risk of lung cancers [e.g., Birk et al. (44)], which tends to support the hypothesis that preventing silicosis would be expected to prevent silica-related lung cancers.

Fortunately, for most workplaces in North America and Europe today, RCS exposures are consistently very low and of much less concern than those characterized in many of the reviewed studies. Therefore, there may be few additional opportunities to identify and follow large numbers of workers highly exposed to RCS to determine the risks of these serious diseases. However, silicosis cases continue to be reported, most recently including manufactured stone installation workers, indicating that uncontrolled exposure scenarios still occur. Additionally, as medical imaging (e.g., computerized tomography or CT scans) and other technologies to assess respiratory health including pulmonary function testing and biological markers of inflammatory responses, modern research questions more likely will be raised regarding the role of RCS exposure and more sensitive indicators of harm to human health than clinical silicosis and lung cancer.
