# Supplementary material for: Systematic review of the epidemiological evidence of associations between quantified occupational exposure to respirable crystalline silica and the risk of silicosis and lung cancer
Source: Front Public Health. 2025 Feb 28;13:1554006. doi: 10.3389/fpubh.2025.1554006 (PMC11906704; doi:10.3389/fpubh.2025.1554006)
Supplement: Supplementary file 1 [file Data_Sheet_1.docx]

Supplementary Material

**List of articles excluded for not meeting minimal study quality ratings for current hypotheses or not reporting at least semi- quantitative exposure-response information (n=34)**

# Allen EM, Alexander BH, MacLehose RF, et al Occupational exposures and lung cancer risk among Minnesota taconite mining workers Occupational and Environmental Medicine 2015;72:633-639.

# Bergdahl IA, Jonsson H, Eriksson K, Damber L, Järvholm B. Lung cancer and exposure to quartz and diesel exhaust in Swedish iron ore miners with concurrent exposure to radon. Occup Environ Med. 2010 Aug;67(8):513-8. doi: 10.1136/oem.2009.047456.

# Brown TP, Rushton L. Mortality in the UK industrial silica sand industry: 2. A retrospective cohort study. Occup Environ Med. 2005 Jul;62(7):446-52. doi: 10.1136/oem.2004.017731.

# Brüske-Hohlfeld I, Möhner M, Pohlabeln H, Ahrens W, Bolm-Audorff U, Kreienbrock L, Kreuzer M, Jahn I, Wichmann HE, Jöckel KH. Occupational lung cancer risk for men in Germany: results from a pooled case-control study. Am J Epidemiol. 2000 Feb 15;151(4):384-95. doi: 10.1093/oxfordjournals.aje.a010218.

# Calvert GM, Rice FL, Boiano JM, Sheehy JW, Sanderson WT. Occupational silica exposure and risk of various diseases: an analysis using death certificates from 27 states of the United States. Occup Environ Med. 2003 Feb;60(2):122-9. doi: 10.1136/oem.60.2.122.

# Carta P, Aru G, Manca P. Mortality from lung cancer among silicotic patients in Sardinia: an update study with 10 more years of follow up. Occup Environ Med. 2001 Dec;58(12):786-93. doi: 10.1136/oem.58.12.786.

# Carta P, Cocco P, Picchiri G. Lung cancer mortality and airways obstruction among metal miners exposed to silica and low levels of radon daughters. Am J Ind Med. 1994 Apr;25(4):489-506. doi: 10.1002/ajim.4700250404.

# Cassidy A, 't Mannetje A, van Tongeren M, Field JK, Zaridze D, Szeszenia-Dabrowska N, Rudnai P, Lissowska J, Fabianova E, Mates D, Bencko V, Foretova L, Janout V, Fevotte J, Fletcher T, Brennan P, Boffetta P. Occupational exposure to crystalline silica and risk of lung cancer: a multicenter case-control study in Europe. Epidemiology. 2007 Jan;18(1):36-43. doi: 10.1097/01.ede.0000248515.28903.3c.

# Chen W, Bochmann F, Sun Y. Effects of work related confounders on the association between silica exposure and lung cancer: a nested case-control study among Chinese miners and pottery workers. Int Arch Occup Environ Health. 2007 Feb;80(4):320-6. doi: 10.1007/s00420-006-0137-0.

# Chen W, Chen J. Nested case-control study of lung cancer in four Chinese tin mines. Occup Environ Med. 2002 Feb;59(2):113-8. doi: 10.1136/oem.59.2.113.

# Chen W, Hnizdo E, Chen JQ, Attfield MD, Gao P, Hearl F, Lu J, Wallace WE. Risk of silicosis in cohorts of Chinese tin and tungsten miners, and pottery workers (I): an epidemiological study. Am J Ind Med. 2005 Jul;48(1):1-9. doi: 10.1002/ajim.20174.

# Chia SE, Chia KS, Phoon WH, Lee HP. Silicosis and lung cancer among Chinese granite workers. Scand J Work Environ Health. 1991 Jun;17(3):170-4. doi: 10.5271/sjweh.1720.

# Cocco P, Rice CH, Chen JQ, McCawley M, McLaughlin JK, Dosemeci M. Non-malignant respiratory diseases and lung cancer among Chinese workers exposed to silica. J Occup Environ Med. 2000 Jun;42(6):639-44. doi: 10.1097/00043764-200006000-00014.

# de Klerk NH, Musk AW. Silica, compensated silicosis, and lung cancer in Western Australian goldminers. Occup Environ Med. 1998 Apr;55(4):243-8. doi: 10.1136/oem.55.4.243.

# Deborah C Glass, Christina Dimitriadis, Jessy Hansen, Ryan F Hoy, Fiona Hore-Lacy, Malcolm R Sim, Silica Exposure Estimates in Artificial Stone Benchtop Fabrication and Adverse Respiratory Outcomes, Annals of Work Exposures and Health, Volume 66, Issue 1, January 2022, Pages 5–13, https://doi.org/10.1093/annweh/wxab044

# Kachuri L, Villeneuve PJ, Parent MÉ, Johnson KC; Canadian Cancer Registries Epidemiology Group; Harris SA. Occupational exposure to crystalline silica and the risk of lung cancer in Canadian men. Int J Cancer. 2014 Jul 1;135(1):138-48. doi: 10.1002/ijc.28629.

# Kleinschmidt SE, Andres KL, Holen BM, Buehrer BD, Durand G, Taiwo O, et al. (2022). Mortality among mine and mill workers exposed to respirable crystalline silica. PLoS ONE 17(10): e0274103. https://doi.org/10.1371/journal.pone.0274103

# Liu Y, Steenland K, Rong Y, Hnizdo E, Huang X, Zhang H, Shi T, Sun Y, Wu T, Chen W. Exposure-response analysis and risk assessment for lung cancer in relationship to silica exposure: a 44-year cohort study of 34,018 workers. Am J Epidemiol. 2013 Nov 1;178(9):1424-33. doi: 10.1093/aje/kwt139.

# Miller BG, MacCalman L. Cause-specific mortality in British coal workers and exposure to respirable dust and quartz. Occup Environ Med. 2010 Apr;67(4):270-6. doi: 10.1136/oem.2009.046151.

# Olsen GW, Andres KL, Johnson RA, Buehrer BD, Holen BM, Morey SZ, Logan PW, Hewett P. Cohort mortality study of roofing granule mine and mill workers. Part II. Epidemiologic analysis, 1945-2004. J Occup Environ Hyg. 2012;9(4):257-68. doi: 10.1080/15459624.2012.667349.

# Picciotto S, Neophytou AM, Brown DM, Checkoway H, Eisen EA, Costello S. Occupational silica exposure and mortality from lung cancer and nonmalignant respiratory disease: G-estimation of structural nested accelerated failure time models. Environ Epidemiol. 2018 Sep;2(3):e029. doi: 10.1097/EE9.0000000000000029.

# Raanan R, Zack O, Ruben M, Perluk I, Moshe S. Occupational Silica Exposure and Dose-Response for Related Disorders-Silicosis, Pulmonary TB, AIDs and Renal Diseases: Results of a 15-Year Israeli Surveillance. Int J Environ Res Public Health. 2022 Nov 15;19(22):15010. doi: 10.3390/ijerph192215010.

# Rice FL, Park R, Stayner L, Smith R, Gilbert S, Checkoway H. Crystalline silica exposure and lung cancer mortality in diatomaceous earth industry workers: a quantitative risk assessment. Occup Environ Med. 2001 Jan;58(1):38-45. doi: 10.1136/oem.58.1.38.

# Romundstad P, Andersen A, Haldorsen T. Cancer incidence among workers in the Norwegian silicon carbide industry. Am J Epidemiol. 2001 May 15;153(10):978-86. doi: 10.1093/aje/153.10.978.

# Sogl M, Taeger D, Pallapies D, Brüning T, Dufey F, Schnelzer M, Straif K, Walsh L, Kreuzer M. Quantitative relationship between silica exposure and lung cancer mortality in German uranium miners, 1946-2003. Br J Cancer. 2012 Sep 25;107(7):1188-94. doi: 10.1038/bjc.2012.374.

# Sun Y, Bochmann F, Morfeld P, Ulm K, Liu Y, Wang H, Yang L, Chen W. Change of exposure response over time and long-term risk of silicosis among a cohort of Chinese pottery workers. Int J Environ Res Public Health. 2011 Jul;8(7):2923-36. doi: 10.3390/ijerph8072923. Epub 2011 Jul 14.

# Ulm K, Waschulzik B, Ehnes H, Guldner K, Thomasson B, Schwebig A, Nuss H. Silica dust and lung cancer in the German stone, quarrying, and ceramics industries: results of a case-control study. Thorax. 1999 Apr;54(4):347-51. doi: 10.1136/thx.54.4.347

# Ulvestad B, Bakke B, Eduard W, Kongerud J, Lund MB. Cumulative exposure to dust causes accelerated decline in lung function in tunnel workers. Occup Environ Med. 2001 Oct;58(10):663-9. doi: 10.1136/oem.58.10.663.

# Ulvestad B, Bakke B, Melbostad E, Fuglerud P, Kongerud J, Lund MB. Increased risk of obstructive pulmonary disease in tunnel workers. Thorax. 2000 Apr;55(4):277-82. doi: 10.1136/thorax.55.4.277.

# Ulvestad B, Ulvestad M, Skaugset NP, Aaløkken TM, Günther A, Clemm T, Lund MB, Ellingsen DG. Pulmonary function and high-resolution computed tomography in outdoor rock drillers exposed to crystalline silica. Occup Environ Med. 2020 Sep;77(9):611-616. doi: 10.1136/oemed-2019-106254.

# Westberg H, Andersson L, Bryngelsson IL, Ngo Y, Ohlson CG. Cancer morbidity and quartz exposure in Swedish iron foundries. Int Arch Occup Environ Health. 2013 Jul;86(5):499-507. doi: 10.1007/s00420-012-0782-4.

# Westberg HB, Bellander T. Epidemiological adaptation of quartz exposure modeling in Swedish aluminum foundries: nested case-control study on lung cancer. Appl Occup Environ Hyg. 2003 Dec;18(12):1006-13. doi: 10.1080/10473220390244676.

# Yu IT, Tse LA, Leung CC, Wong TW, Tam CM, Chan AC. Lung cancer mortality among silicotic workers in Hong Kong--no evidence for a link. Ann Oncol. 2007 Jun;18(6):1056-63. doi: 10.1093/annonc/mdm089.

# Zeka A, Mannetje A, Zaridze D, Szeszenia-Dabrowska N, Rudnai P, Lissowska J, Fabiánová E, Mates D, Bencko V, Navratilova M, Cassidy A, Janout V, Travier N, Fevotte J, Fletcher T, Brennan P, Boffetta P. Lung cancer and occupation in nonsmokers: a multicenter case-control study in Europe. Epidemiology. 2006 Nov;17(6):615-23. doi: 10.1097/01.ede.0000239582.92495.b5.

# Supplemental Table S1: Summary of the RCS exposure metrics and the reported risk estimates for smoking in the silicosis studies.

| **Study**   **(First Author Year)** | **Cumulative Exposure** | **Mean Exposure Concentration** | **Employment Duration** | **Smoking** |
| --- | --- | --- | --- | --- |
| Cherry 1998 | OR=1.37 (1.24-1.53)  Per 1 mg/m^3^-yr | OR=2.66 (1.94-3.66)  Per 0.1 mg/m^3^ | OR=1.08 (0.83-1.40)  Per 10 yr | OR=2.28 (1.02-5.10) |
| Hughes 1998  *(overlaps with Park 2002)*  *Park 2002* | Concentration <0.50 mg/m3  Cumulative mg/m^3^-yr), RR  < 1 1.00 >1, <3 0.96 (0.26-3.6) >3, < 6 3.88 (1.22-12.4) >6 4.34 (1.06-8.4)  RR=5.93 at 1 mg/m^3^-yr using best fit linear relative rate model | Concentration >0.50 mg/m3  Cumulative mg/m^3^-yr), RR  < 1 1.00 >1,< 3 11.8 (1.55-89.2) >3,< 6 28.1 (3.8-207.9) >6 20.1 (2.7-151.4)  RR=25.64 at mean exposure | Not Reported  Not Reported | Workers with <0.50 mg/m3 mean exposure, smoking was significantly related to opacities (0.4% (1 of 269) of nonsmokers developing opacities compared to 2.6% (20 of 756) of smokers (one-tailed p < 0.01)  Not reported for silicosis analysis, but considered a negative confounder for LDOC analyses (lung disease other than cancer) |
| Lenander-Ramirez 2022 | >0.39 mg/m3-yr SIR=45.87 (16.83–99.83)  (No cases below 0.39) | Not Reported | Not Reported | Not Reported |
| Birk 2025 (ILO >1/1 results) | Cumulative (mg/m^3^-yr), HR  ≤0.5 Reference *>*0.5–1.0 0.9 (0.1–5.5) *>*1.0–1.5 1.3 (0.2–8.1) *>*1.5–3.0 0.8 (0.1–5.1) *>*3.0-4.0 3.3 (0.6–18.1) >4.0-5.0 6.5 (1.3-32.4) >5.0-6.0 10.5 (2.4-46.2) >6.0 9.2 (2.3-36.8) | Avg. Exposure (mg/m^3^), HR  ≤0.05 Reference *>*0.05–0.1 0.9 (0.2–4.7) *>*0.1–0.15 4.0 (1.1–15.2) *>*0.15–0.2 11.1 (3.6–34.4) *>* 0.2 20.0 (7.5–53.1) | Yrs employed, HR  ≤10 Reference *>*10–20 0.8 (0.2–3.5) *>*20–30 1.7 (0.4–7.0) *>*30 1.2 (0.3–4.7) | Smoking status, HR  Never Reference Ever 2.4 (1.2–4.9) Unknown 0.6 (0.3–1.4) |
| Rego 2008 | 1.35 (1.18 –1.54) Per 1 mg/m^3^-yr | Not Reported | 1.02 (0.95–1.11) per work-yr | 1.00 (0.96 –1.05) per packyear |
| Steenland 2001 | Cumulative (mg/m^3^-yr), SRR (cases)  >0–0.10 1.00 (1) >0.10–0.51 1.22 (2) >0.51–1.28 2.91 (4) >1.28 7.39 (7) | Not Reported | Not Reported | Not Reported |
| Vacek 2019  *(overlaps with Hughes 2001; McDonald 2005)* | OR=1.43 (1.23–1.66) per 1 mg/m^3^-years | OR=1.30 (1.11–1.51) per 0.10 mg/m^3^ | OR=1.10 (1.05–1.16) per year (net exposure duration) | Not Reported |
| Vacek 2011 | OR=1.13 (1.05 to 1.21) per 1 mg/m^3^-year | OR=1.50 (1.13 to 1.98) per 0.10 mg/m^3^ | OR=1.36 (1.06 to 1.76) per 10 years work (net duration of exposure) | Not Reported |
| Wang 2020a  *(overlaps with Lai 2018; Chen 2012; Chen 2001; Chen 1992)* | HR=1.12 (1.11-1.12) per 1 mg/m^3^-year (overall for all cohorts) | Not Reported | Only presented graphically | <1.81 mg/m^3^-years ever smoking HR=1.14 (0.98-1.33)  > 1.81 mg/m^3^-years ever smoking HR=5.79 (5.01-6.69) |
| Zhang 2010 | OR=5.38 (3.82-7.57) per 1 mg/m^3^-year | Not Reported | RR= 10.04 (2.42-41.73) for >30 years work | OR=4.79 (2.24-10.27) |

# Supplemental Table S2: Summary of the RCS exposure metrics and the reported risk estimates for smoking in the lung cancer studies.

| **Study**   **(First Author Year)** | **Cumulative Exposure** | **Mean Exposure Concentration** | **Employment Duration** | **Smoking** |
| --- | --- | --- | --- | --- |
| Hnizdo 1997 | Cumulative (mg/m^3^-yr), RR (95% CI)  2.7-4.3 1.83 (0.8-4.1)  4.4-6.3 1.85 (0.8-4.3)  >6.3 3.19 (1.3-7.6) | Not Reported | Net year in dusty conditions lagged by 20 yrs from death, RR (95% CI)  10-15 1.63 (0.6-4.6)  16-20 2.07 (0.7-6.3)  >20 3.36 (1.02-10.7) | Model 1  Smoking packyears, RR (95% CI)  6.5-20 2.9 (0.8-11.0)  21-30 4.9 (1.4-17.0)  >30 10.1 (3.1-33.6) |
| Steenland 2001 | Cumulative (mg/m^3^-yr) (15 yr lag), OR (95% CI)  0–0.18 1.00  >0.18–0.59 1.35 (0.72-2.54)  >0.59–1.23 1.63 (0.83-3.18)  >1.23 2.00 (1.00-4.01)  (p-value for trend=0.08) | Quartiles (mg/m^3^) in  nested case-control, OR (95% CI)  0-0.023 1.00  >0.023-0.046 0.92 (0.42-2.00)  >0.046-0.065 1.44 (0.72-2.86)  >0.065 2.26 (1.17-4.38) | Months/years, SMR (95% CI)  <6 months 2.38 (1.17-4.22)  6 mo.-1yr 1.67 (0.54-3.90)  1-2 yrs 2.27 (0.91-4.68)  2-5 yrs 1.11 (p.44-2.29)  5-10 yrs 0.84 (0.27-1.96)  10-20 yrs 1.67 (0.99-2.64)  >20 yrs 1.54 (1.05-2.17) | Not Reported |
| McDonald 2005 | Cumulative (mg/m3-yr) (15 yr lag), OR (cases)  ≤0.3 1.00 (13)  >0.3- ≤1.1 0.94 (17)  >1.1-≤3.3 2.24 (38)  >3.3 2.66 (37) | Avg. Exposure (mg/m^3^), OR  ≤0.07 Reference  *>*0.07–0.16 1.01 (0.48–2.12)  *>*0.16–0.26 1.62 (0.75–3.53)  *>* 0.26 2.36 (1.00–5.59) | Yrs employed, OR  ≤12 1.00  *>*12–20 1.53  *>*20–32 0.62  *>*32 0.63 | Smoking status, OR  Never 1.00  Ever 5.37 (2.33–12.35) |
| Pukkala 2005 | Cumulative (mg/m^3^-yr), RR  (20 yr lag)  ≤0.9 1.05 (1.00-1.10)  1.0–9.9 0.97 (0.91-1.03)  ≥10 1.42 (1.20-1.70) | Not Reported | Not Reported | Not Reported |
| Preller 2010 | Cumulative (mg/m^3^-yr), RR  >0 to <3 0.95 (0.73-1.25)  ≥3 1.47 (0.93-2.33) | Mean Concentration (mg/m^3^), RR  >0-<0.075 0.97 (0.70-1.33)  *0*.075-0.2 1.21 (0.82–1.78)  0.2-0.6 1.14 (0.63-2.05) | Years of exposure, RR  1-10 0.67 (0.43-1.04)  11-25 0.88 (0.60–1.29)  26-51 1.65 (1.14–2.41) | Not Reported |
| Birk 2025 | Cumulative (mg/m^3^-yr), HR  Men  ≤0.5 Reference *>*0.5–1.0 0.8 (0.5–1.3) *>*1.0–1.5 0.8 (0.5–1.4) *>*1.5–3.0 0.9 (0.6–1.5) *>*3.0-4.0 1.2 (0.7–2.3) >4.0-5.0 1.2 (0.6-2.4) >5.0-6.0 1.5 (0.8-2.8) >6.0 1.0 (0.6-1.6)  Women  ≤0.5 Reference *>*0.5–1.0 1.0 (0.6–1.8) *>*1.0–1.5 0.5 (0.2–1.2) *>*1.5–3.0 0.7 (0.4–1.4) *>*3.0-4.0 1.1 (0.5–2.6) >4.0-5.0 0.8 (0.3-2.2) >5.0-6.0 0.5 (0.1-2.0) >6.0 0.6 (0.2-1.7) | Avg. Exposure (mg/m^3^), HR  Men  ≤0.05 Reference *>*0.05–0.1 1.5 (1.0–2.3) *>*0.1–0.15 1.2 (0.7–2.1) *>*0.15–0.2 2.2 (1.4–3.6) *>* 0.2 1.3 (0.8–2.2)  Women  ≤0.05 Reference *>*0.05–0.1 0.4 (0.2–0.9) *>*0.1–0.15 0.9 (0.4–1.7) *>*0.15–0.2 0.5 (0.2–1.3) *>* 0.2 0.4 (0.1-2.7) | Yrs employed, HR  Men  ≤10 Reference *>*10–20 0.7 (0.4–1.2) *>*20–30 0.7 (0.4–1.1) *>*30 0.7 (0.5–1.2)  Women  ≤10 Reference *>*10–20 0.7 (0.4–1.5) *>*20–30 0.7 (0.4–1.3) *>*30 0.7 (0.4–1.4) | Smoking status, HR  Men  Never 1.0 Ever 17.9 (7.3–43.7) Unknown 7.5 (3.0–18.6)  Women  Never 1.0 Ever 6.1 (3.4-10.9) Unknown 1.6 (0.9-2.9) |
| Bugge 2012 | Cumulative (mg/m^3^-yr), SIR (20 yr lag)  0-0.026 1.4 (1.0-2.0)  0.026-0.077 1.8 (1.1-3.0)  0.077-2.3 2.0 (1.3-3.3) | Not Reported | Not Reported | Smoking, IRR  Never 1.0  Ever 20.9 (2.9-151)  Unknown 7.6 (0.8-73) |
| Cherry 2013 | “In additional analyses (details not shown) cumulative exposure was unrelated to any of the outcomes of interest in either period.” | Mean Exposure (mg/m^3^), HR  <0.1 1.00  0.1-<0.15 1.07 (0.65-1.74)  0.15-<0.2 0.76 (0.43-1.32)  ≥0.2 0.96 (0.58-1.60) | Duration of employment (yrs), HR  <2.5 1.00  2.5-<10 1.43 (0.97-2.09)  10-<25 0.98 (0.63-1.52)  >25 1.06 (0.68-1.64) | Not Reported |
| Graber 2014 | Cumulative (mg/m^3^-yr), HR  <2.22 1.0  2.22–3.30 1.08 (0.85-1.37)  3.31–4.12 1.20 (0.95-1.52)  ≥4.13 1.17 (0.92-1.50) | Not Reported | Not Reported | Smoking, HR  Never 1.0  Former 2.70 (1.51-4.83)  Current 9.27 (5.33-16.11) |
| Gallagher 2015 | Cumulative (mg/m^3^-yr), HR  (15 yr lag)  <0.4 1.00  0.4–<0.9 1.38 (0.76-2.52)  1.0–<2.6 1.24 (0.73-2.10)  2.6-<5.6 1.98 (1.11-3.54)  >5.6 2.36 (1.25-4.46) | Not Reported | Not Reported | Not Reported |
| Ge 2020 | Cumulative (mg/m^3^-yr), OR  Men – all cases  >0–0.39 1.15 (1.04-1.27)  0.4–1.09 1.33 (1.21-1.47)  1.1–2.39 1.29 (1.17-1.42)  ≥2.4 1.45 (1.31-1.60)  Men - Never smokers only  >0–0.39 1.17 (0.85-1.57)  0.4–1.09 1.07 (0.78-1.43)  1.1–2.39 1.02 (0.75-1.36)  ≥2.4 1.40 (1.03-1.86)  Women – all cases  >0–0.39 1.07 (0.77-1.48)  0.4–1.09 1.24 (0.88-1.74)  1.1–2.39 1.02 (0.69-1.47)  ≥2.4 1.10 (0.69-1.74) | Not Reported | Duration (yrs), OR  Men  1-9 1.22 (1.12-1.31)  10-19 1.20 (1.08-1.34)  20-29 1.45 (1.26-1.66)  >29 1.48 (1.34-1.63)  Women  1-9 1.08 (0.84-1.39)  10-19 1.13 (0.75-1.69)  20-29 1.40 (0.74-2.63)  >29 1.05 (0.67-1.63) | Men Ever-smoker and never silica OR=6.37 (5.91-6.87)  Men Ever-smoker and ever-silica OR=8.72 (8.0-9.52)  Men - SYNERGY study (Pesch et al. 2012) Former smokers OR=7.5 (6.5-8.7) Current smokers OR=23.6 (20.4-27.2)  Women - SYNERGY study (Pesch et al. 2012) Former smokers OR=2.8 (2.4-3.3) Current smokers OR=7.8 (6.8-9.0) |
| Wang 2020 | Cumulative (mg/m^3^-yr), HR  0 to 1.056 1.32 (1.07-1.62)  1.057-3.925 1.51 (1.25-1.83)  >3.925 1.52 (1.24-1.87)  Never-smoked HR=1.43 (0.99-2.08) | Not Reported | Not Reported | Ever-smoker and never silica HR=3.32 (2.34-4.71)  Ever-smoker and ever-silica HR=4.51 (3.23-6.29) |

# Supplemental Figure S1. Tiering approach used in evaluating epidemiological studies based on modified National Toxicology Program (NTP) Office of Health Assessment and Translation (OHAT) framework.

**Tier 1:** Quantitative Exposure Characterization

Low Quality
(e.g, no control for relevant occupational co-exposures or smoking)

**Tier 3:** Remaining TSCA Key Criteria

- Participant Selection
- Outcome Measurement
- Additional adjustments (race, sex, etc.)
- Study Design & Methods

High Quality
(most of the remaining criteria accounted for in the study)

Medium Quality
(not all remaining criteria accounted for in the study)

Not Relied Upon for Risk Evaluation

**Tier 4:** Exposure-response Information Available

Relied Upon for Risk Evaluation

**Tier 2:** Control of Confounders

Exclude
(qualitative only e.g. by job title)

YES

YES

YES

NO

NO

NO

NO

YES

**Supplemental Figure S2. Heat Map of Study Quality by Domain for Silicosis Exposure-Response Studies.**

| **Author/Year** | **Cherry 1998** | **Hughes 1998** | **Steenland 2001** | **Rego 2008** | **Zhang 2010** | **Vacek 2011** | **Vacek 2019** | **Wang 2020a** | **Lenander-Ramirez 2022** | **Birk 2025** |
| --- | --- | --- | --- | --- | --- | --- | --- | --- | --- | --- |
| **Domain I (Study Participation)** | H | M | M | L | M | M | M | M | L | H |
| **Domain II (Exposure Characterization)** | M | M | M | M | M | M | M | M | H | M |
| **Domain III (Outcome Assessment)** | L | M | M | M | M | M | M | M | M | H |
| **Domain IV (Potential Confounding/Variable Control)** | M | L | L | M | L | L | L | L | M | M |
| **Domain V (Analysis)** | M | M | M | M | M | M | M | M | M | M |
| **Overall Rating** | M | M | M | M | M | M | M | M | M | H |

**Supplemental Figure S3. Heat Map of Study Quality by Domain for Lung Cancer Exposure-Response Studies.**

| **Author/Year** | **Hnizdo 1997** | **Steenland 2001** | **McDonald 2005** | **Pukkala 2005** | **Preller 2010** | **Bugge 2012** | **Cherry 2013** | **Graber 2014** | **Gallagher 2015** | **Ge 2020** | **Wang 2020b** | **Birk 2025** |
| --- | --- | --- | --- | --- | --- | --- | --- | --- | --- | --- | --- | --- |
| **Domain I (Study Participation)** | H | M | M | M | M | M | M | M | M | M | M | H |
| **Domain II (Exposure Characterization)** | L | M | M | M | L | M | M | M | M | M | L | M |
| **Domain III (Outcome Assessment)** | M | M | M | H | M | H | M | M | M | L | M | M |
| **Domain IV (Potential Confounding/Variable Control)** | L | L | L | M | M | M | L | M | M | M | M | M |
| **Domain V (Analysis)** | M | M | M | M | M | M | M | M | M | M | M | M |
| **Overall Rating** | M | M | M | M | M | M | M | M | M | M | M | M |
